# Potential Anti-Cholinesterase Activity of Bioactive Compounds Extracted from *Cassia grandis* L.f. and *Cassia timoriensis* DC.

**DOI:** 10.3390/plants12020344

**Published:** 2023-01-11

**Authors:** Maram B. Alhawarri, Roza Dianita, Mira Syahfriena Amir Rawa, Toshihiko Nogawa, Habibah A. Wahab

**Affiliations:** 1 School of Pharmaceutical Sciences, Universiti Sains Malaysia, Minden 11800, Malaysia; 2 Faculty of Pharmacy, Jadara University, Irbid 21110, Jordan; 3 USM-RIKEN Interdisciplinary Collaboration for Advanced Sciences (URICAS), Universiti Sains Malaysia, Gelugor 11800, Malaysia; 4 Molecular Structure Characterization Unit, Technology Platform Division, RIKEN Center for Sustainable Resource Science, 2-1 Hirosawa, Saitama 351-0198, Japan

**Keywords:** *Cassia timoriensis*, *Cassia grandis*, Alzheimer’s disease, acetylcholinesterase, butyrylcholinesterase, molecular docking

## Abstract

Acetylcholinesterase (AChE) inhibitors remain the primary therapeutic drug that can alleviate Alzheimer’s disease’s (AD) symptoms. Several *Cassia* species have been shown to exert significant anti-AChE activity, which can be an alternative remedy for AD. *Cassia timoriensis* and *Cassia grandis* are potential plants with anti-AChE activity, but their phytochemical investigation is yet to be further conducted. The aims of this study were to identify the phytoconstituents of *C. timoriensis* and *C. grandis* and evaluate their inhibitory activity against AChE and butyrylcholinesterase (BChE). Two compounds were isolated for the first time from *C. timoriensis*: arachidyl arachidate (**1**) and luteolin (**2**). Five compounds were identified from *C. grandis*: β-sitosterol (**3**), stigmasterol (**4**), cinnamic acid (**5**), 4-hydroxycinnamic acid (**6**), and hydroxymethylfurfural (**7**). Compound **2** showed significant inhibition towards AChE (IC_50_: 20.47 ± 1.10 µM) and BChE (IC_50_: 46.15 ± 2.20 µM), followed by **5** (IC_50:_ 40.5 ± 1.28 and 373.1 ± 16.4 µM) and **6** (IC_50_: 43.4 ± 0.61 and 409.17 ± 14.80 µM) against AChE and BChE, respectively. The other compounds exhibited poor to slightly moderate AChE inhibitory activity. Molecular docking revealed that **2** showed good binding affinity towards *Tc*AChE (PDB ID: 1W6R) and *Hs*BChE (PDB ID: 4BDS). It formed a hydrogen bond with TYR121 at the peripheral anionic site (PAS, 2.04 Å), along with hydrophobic interactions with the anionic site and PAS (TRP84 and TYR121, respectively). Additionally, **2** formed three H-bonds with the binding site residues: one bond with catalytic triad, HIS438 at distance 2.05 Å, and the other two H-bonds with GLY115 and GLU197 at distances of 2.74 Å and 2.19 Å, respectively. The evidence of molecular interactions of **2** may justify the relevance of *C. timoriensis* as a cholinesterase inhibitor, having more promising activity than *C. grandis*.

## 1. Introduction

Alzheimer’s disease (AD) is a neurodegenerative disorder, characterized by progressive memory loss along with cognitive and learning insufficiencies [1]. AD was described for the first time by a German psychiatrist Alois Alzheimer in 1901 and named as “Alzheimer’s disease” in 1910 by Dr. Kraeplin [2]. Around 50 million people worldwide are living with AD, and this number of individuals is expected to triple to around 152 million by 2050 [3,4]. Despite vast research towards AD globally, its etiology and pathogenesis remain unclear [5]. However, in the last few decades, many studies have proposed that the pathogenesis of AD may be linked to various hypotheses, including the cholinergic hypothesis, beta-amyloid (Aβ) hypothesis, tau hypothesis, and neuroinflammatory hypothesis [6]. The cholinergic hypothesis is the most extensively investigated theory and has been directly associated with AD in recent studies [7]. It is characterized by the depletion of acetylcholine (ACh) around the cholinergic synaptic neurons in the cortex as compared to the healthy brain [8,9]. Cholinesterase inhibitors improve cognition and memory loss in AD patients by preventing ACh from being degraded by acetylcholinesterase (AChE) and butyrylcholinesterase (BChE) [10,11]. AChE is a potent catalyzer capable of hydrolyzing roughly 250,000 molecules of ACh per second and is more strongly linked to cognitive function than BChE [12,13]. Therefore, the most frequently held belief in AD therapy is that AChE inhibitors can improve cognitive function by increasing ACh-mediated neuronal transmission [14]. AChE inhibitors, which had previously been used to treat myasthenia gravis, were utilized as the first FDA-approved drugs for AD [15,16]. In contrast, BChE, which is found in considerably lower amounts, is referred to as a “pseudo-cholinesterase” and is thought to have more restricted distribution in the brain [17]. Nevertheless, BChE activity increases while AChE activity declines as dementia symptoms progress [18]. Therefore, BChE inhibition may also be advantageous in the late stages of AD [19,20].

Historically, a range of acetylcholinesterase inhibitors (AChEIs) have been investigated as potential candidates for the symptomatic treatment of AD, including naturally occurring chemicals such as physostigmine, huperzine A, and galantamine, as well as synthetic and semi-synthetic compounds such as tacrine, donepezil, and rivastigmine [10,21]. Among various medicinal plants studied, *Cassia* species have been explored as a potential therapeutic adjuvant for AD. For example, *Cassia tora* Linn has been widely studied for its multifunctional anti-AD potential [22,23,24,25]. In vitro studies revealed that *C. tora* extract inhibits the aggregation of Aβ(1–42) oligomers and helps in the dissociation of the pre-formed Aβ fibrils [22,25]. An in vivo study demonstrated the beneficial effects of *C. tora* extract in a rat model in lowering oxidative stress in the hippocampus and cortex [23]. Decreased levels in lipid peroxidation, proinflammatory mediators, primarily cytokines, and AChE activity were observed [23]. This is indicative of *C. tora* extract being capable of ameliorating cognitive impairment and AD symptoms [24]. *Cassia obtusifolia* seed extract also improves memory impairment in a mouse model via the anti-AChE capacity [26].

Our previous study screened 17 methanolic extracts from five different *Cassia* species, namely *Cassia fistula*, *Cassia alata*, *Cassia spectabilis*, *Cassia timoriensis*, and *Cassia grandis* [27]. *C. timoriensis* and *C. grandis* demonstrated the strongest anti-AChE potential [27]. As a follow-up to our current research to explore more natural bioactive compounds exerting anti-cholinesterase activity, the present study was conducted to investigate the phytochemical constituents of *C. timoriensis* flowers and *C. grandis* pods and determine their inhibitory potential against cholinesterase via in vitro and in silico approaches.

## 2. Results and Discussion

### 2.1. In Vitro Screening for Acetylcholinesterase Inhibition of C. timoriensis and C. grandis Extracts

Our previous screening of 17 methanolic extracts from *C. fistula, C. alata, C. spectabilis, C. timoriensis,* and *C. grandis* showed that *C. timoriensis* and *C. grandis* exhibited promising AChE inhibitory activity [27]. Additionally, our group analyzed different extracts of *C. timoriensis* flowers for phytochemical analysis and inhibitory activity [28]. It was found that ethyl acetate, methanol, and hexane extract of *C. timoriensis* with IC_50_ values of 6.91, 6.40, and 12.08 µg/mL, respectively, were a promising option for further chemical investigation [28]. Based on the previous AChE screening results along with TLC profiling, only *n*-hexane and ethyl acetate extracts were chosen for further chemical analysis and purification. Few studies have been carried out towards *C. grandis* and *C. timoriensis* related to AD and anti-cholinesterase activity.

Furthermore, the AChE inhibitory activity of *C. grandis* pods was evaluated in this study, as shown in Table 1. Methanol and ethyl acetate extracts of *C. grandis* pods revealed the greatest inhibitory activity, with IC_50_ values of 74.09 ± 1.60 and 82.96 ± 2.81 µg/mL, respectively (Table 1). It was also noticed that the aqueous and hexane fractions had negligible inhibitory efficacy on AChE. Thus, based on the screening results for *C. grandis*, ethyl acetate and methanol extracts were selected for further chemical investigation and an isolation process.

### 2.2. Liquid Chromatography–Mass Spectrometry Analysis of C. timoriensis and C. grandis

**Figure 1:**
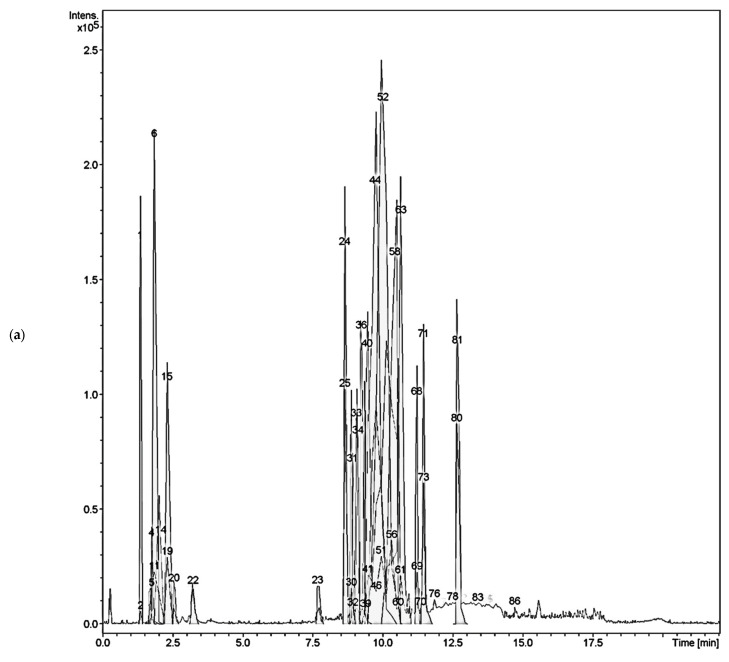

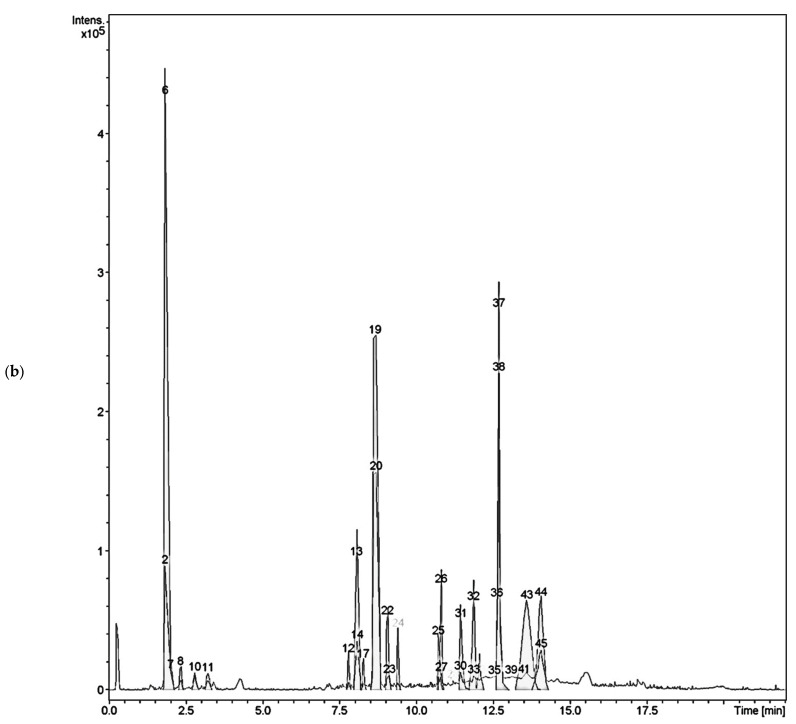
LC-MS/MS chromatograms of the aqueous ethanolic extracts of (**a**) *Cassia timoriensis*, (**b**) *Cassia grandis*.

Insufficient research was found on the phytochemical content as well as the anti-cholinesterase activity of *C. timoriensis* and *C. grandis*. In addition, higher inhibitory activity was found in the *C. timoriensis* extract when compared to the isolated components. Hence, the aqueous ethanolic extracts of both plants were analyzed using a liquid chromatography–time of flight–mass spectrometry, LC-TOF-MS (Bruker/MicroTOF QIII) instrument in order to profile their chemical constituents and to identify the principal components in each plant extract that could contribute to their cholinesterase inhibitory activity. For LCMS analysis, the aqueous ethanolic extracts were used to profile the secondary metabolites of the plant. As a solvent, 80% aqueous ethanol was chosen for its reported ability to extract a wide range of phytochemicals [29,30]. The LC-MS/MS chromatograms of both plants are shown in Figure 1.

The identification of the chemical constituents of each plant was carried out using the available servers and websites (Massbank (https://massbank.eu/MassBank/, accessed on 15 December 2021), MetFrag (https://msbi.ipb-halle.de/MetFrag/, accessed on 20 December 2021), Metline(https://metlin.scripps.edu/, accessed on 1 November 2021), MoNA (https://mona.fiehnlab.ucdavis.edu/, accessed on 1 January 2022), and CFM-ID(https://cfmid.wishartlab.com/, accessed on 1 November 2021), along with literature data comparison [31,32]. A total of 24 components were found and tentatively identified from *C. timoriensis*, with the majority of them belonging to the phenolic acid and flavonoid classes. Retention times (Rts), exact molecular weights, molecular formulas, and the main fragment ions of each identified peak are summarized in Table 2. The metabolites that eluted in the first two minutes of the analysis were mainly saccharide moieties such as lactose, D-glucose, and D-mannose. Quinic acid (phenolic acid) was also found at a retention time of 2.3 min and *m*/*z* 191.0567. Chemicals detected between 7 and 13 min of analysis time were mainly identified as flavonoids. LC-MS/MS detection and literature data comparison of *Cassia* species particularly demonstrated the presence of nearly all types of flavonoids, including flavones (luteolin and kaempferol) [33], flavonols (quercetin) [34,35], isoflavones (chrysoeriol) [36], flavan-3-ols (catechin-3-rhamnoside) [37], cyanidin 3-(6″-benzoyl) glucoside, as well as biflavonoids (2″,3″-dihydro-5′,6″-biluteolin and epiafzelechin-(4β→8)2-epiafzelechin) and triflavonoids (*ent*-fisetinidol-(4β→8)-catechin-(6→4β)-*ent*-fisetinidol) [38,39,40]. It is worth noting that these chemicals were reported for the first time in *C. timoriensis* using UPLC-TOF/QIII-MS analysis.

In comparison to *C. timoriensis*, the LC-MS/MS chromatogram of *C. grandis* demonstrated a lower detectable components content. During a total run time of 22 min, seventeen metabolites were identified using LC-MS/MS analysis of *C. grandis* ethanolic extracts. The sugar moieties were predicted for nine of the examined peaks in the LC-MS/MS chromatogram (Figure 1, Table 3), including monosaccharide sugar (mannose), disaccharide (lactose and sucrose), and oligosaccharide at *m*/*z* 683.2264 and 471.1718. A previous phytochemical investigation revealed a high saccharide content in *C. grandis* pod extracts [41]. Numerous previous studies also confirmed the presence of galactomannans in *C. grandis*, a polysaccharide with a mannose backbone, which confirmed our LC-MS/MS results (Table 3) [42,43,44]. The LC-MS/MS analysis also detected trans-cinnamic acid at *m*/*z* 147.0450, which corresponded to the isolation result. Additionally, luteolin glucoside and kaempferol were detected at *m*/*z* 447.0932 and 285.0402, respectively (Table 3) [45]. However, peak fragmentation analysis demonstrated that the flavonoid content in *C. grandis* was lower than in *C. timoriensis*, which might justify the higher inhibitory activity of *C. timoriensis* extract against AChE and BChE compared to *C. grandis*.

### 2.3. Identification of Isolated Compounds

**Figure 2:**
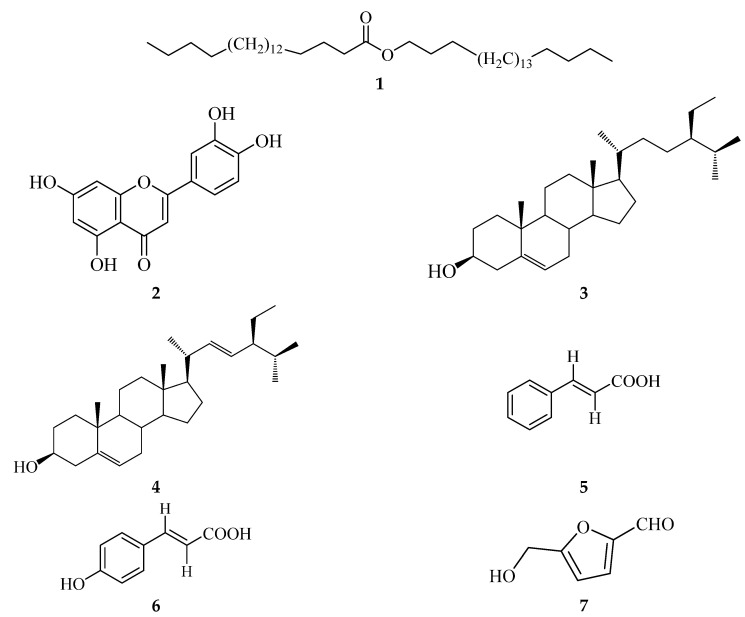
The chemical structure of the isolated compounds of *C. timoriensis* flowers and *C. grandis* pods.

Our previous study on the leaves and flowers of *C. timoriensis* resulted in the isolation of five compounds, 3-methoxyquercetin, β-sitosterol, stigmasterol, octadecanol, and benzene-propanoic acid [27]. In addition, as a follow-up to the previous study, the *n*-hexane and ethanol extracts of *C. timoriensis* flowers were subjected to multiple rounds of purification (Figure S1), yielding two compounds. The *n*-hexane extract yielded 46.2 mg of arachidyl arachidate (**1**), whereas the ethyl acetate extract afforded 21.5 mg of the known flavonoid luteolin (**2**) (Figure 2). Meanwhile, the methanol and ethyl acetate extracts of *C. grandis* were subjected to several rounds of purification steps (Figure S1) to yield five compounds. The ethyl acetate extract yielded 51.8 mg of a mixture of the known sterols β-sitosterol (**3**) and stigmasterol (**4**), 398 mg of trans-cinnamic acid (**5**), and 16.1 mg of p-hydroxycinnamic acid (**6**), while the hydrolyzed methanol extract yielded 90.1 mg of hydroxymethylfurfural (**7**) and also 52.6 mg of a mixture of **3** and **4** (Figure 2).

Analysis of the spectral data of compound **1** showed distinctive peaks of long-chain fatty acid ester (Figures S2 and S3). However, there is no information available in the literature about the spectrum data for this compound. The obtained data were compared to the related structure, arachidic acid fatty acid [46]. Hence, according to the NMR inspection, mass data, and literature data, the structure of **1** was confirmed as arachidyl arachidate, which is known as a one of the natural waxes found in plants and microorganisms [47,48].

The spectroscopic and spectrometric analyses of **2** indicated that is a tetrahydroxyflavone derivative with structural similarities to the well-known flavonoid quercetin. According to the NMR inspection, mass data, and literature data [33,49,50], the structure of compound **2** was confirmed as the known flavonoid luteolin (Figure S4). Compound **2** has been isolated and discovered in a broad variety of *Cassia* species, such as *C. uniflora* [49], *C. mimosoides* [50], and *C. alata* [33]. Compounds **3** and **4** were obtained as a mixture of white needles from *C. grandis* pods. According to the NMR, MS, and literature data [51,52,53,54], the white crystals were identified as a mixture of β-sitosterol (**3**) and stigmasterol (**4**) (Figure S5), which was isolated and identified as a mixture also from the tubers of *C. sieberiana* [51]. Furthermore, **3** and **4** mixtures were reported from *C. timoriensis* flowers in our previous work [27], indicating the broad presence of these phytosterol in *Cassia* genus.

The analysis of the spectroscopic and spectrometric data of **5** along with literature data comparison indicated that this compound is referred to as trans-cinnamic acid (Figure S6) [55]. In addition, the analysis of **6** showed a structure similarity to **5** with an extra hydroxyl group, as indicated by ^1^H and ^13^C NMR data, consistent with the 16 amu difference in the MS data of **6** relative to **5**, indicating that **6** is 4-hydroxycinnamic acid (p-coumaric acid), as supported by literature data (Figure S7) [56]. Compound **7** was identified as hydroxymethylfurfural on the basis of an analysis of the spectral data and literature data comparison (Figure S8) [57].

To the best of our knowledge, this is the first time **1** and **2** have been identified from *C. timoriensis,* while **4**, **5**, and **6** were reported for the first time from *C. grandis* pods.

### 2.4. In Vitro Cholinesterase Activity of Isolated Compounds

The cholinesterase inhibitory potentials of the isolated compounds were evaluated via a spectrophotometric approach, which was adapted by Ellman’s method [58]. The potential of isolated compounds to inhibit AChE and BChE in vitro were tested using galantamine as a positive control. The results reported as IC_50_ values are described in Table 4. Enzyme inhibition is a beneficial approach in order to evaluate the pharmacological potential of herbal remedies and pure substances. The inhibition of the AChE and BChE enzymes has been linked to the alleviation of symptoms associated with memory impairment in AD [10]. Compound **2** demonstrated the highest anti-AChE activity among the isolated compounds, followed by **5**, **6,** and **7** with IC_50_ values of 20.47 ± 1.10, 40.50 ± 1.28, 43.40 ± 0.61, and 158.04 ± 3.49 µM, respectively. Meanwhile, the mixture **3** and **4** demonstrated a moderate to weak inhibition of AChE with an IC_50_ of 78.44 ± 0.70 µg/mL for AChE and 87.29 ± 3.61 µg/mL for BuChE. In addition, compound **2** also had the highest inhibitory potential against BChE, with an IC_50_ of 46.15 ± 2.20 µM, followed by compounds **5** and **6**, which had roughly similar anti-BChE potentials. (Table 4). Previous research indicated that **2** had strong cholinesterase inhibitory activity [59,60], which is similar to our finding in Table 4. The reported AChE inhibition potential of luteolin isolated from *Rhizoma drynariae* was 17.26 ± 0.23 µM [59]. Similarly, luteolin isolated from *Globularia meridionalis* demonstrated IC_50_ values of 25.2 ± 0.4 µg/mL for AChE and of 37.2 ± 0.5 µg/mL for BuChE [60]. Aside from its cholinesterase activity, luteolin protects against Alzheimer’s disease through lowering neuroinflammatory reactions [61]. Previous research has shown that luteolin has a neuroprotective effect by lowering neuroinflammatory reactions [62]. It has been shown to inhibit macrophage/monocytes, T cells, and mast cells, as well as decrease the release of inflammatory mediators [62]. In vivo, luteolin protects against hydrogen peroxide (H_2_O_2_), nitric oxide (NO), and malondialdehyde (MDA), as well as restoring acetylcholinesterase, glutathione S-transferase, and superoxide dismutase (SOD) activities [63]. Comparing **2** to other flavonoids from the literature, flavonoids with more hydroxyl groups exhibited a greater inhibition on AChE [64]. Methoxylation may decrease or increase activity depending on the type of flavonoids (flavones, flavanones, or isoflavones) [64]. Glycosylation significantly diminishes flavonoids’ AChE inhibitory action and affinity for AChE by a factor of 1 to 5, depending on the connection site and sugar moiety. In comparison to **2**, which contains hydroxyl groups at positions 6, 8, 3’, and 4’, apigenin (absence of OH group at position 4′) had an IC_50_ of 34.43 ± 2.41 µM (AChE) [65], baicalein (absence of OH groups at position 3′ and 4′) had an IC_50_ of 45.95 ± 3.44 µM (AChE) [66], and acacetin (absence of OH group at position 4′, and methoxylation at position 4′) had an IC_50_ of 65.3 µM [67]. Thus, all these alterations demonstrated that these inhibitors are less active than **2**. Moreover, the hydrogenation of the double bond next to the carbonyl carbon in the structure of flavanones also decreases their affinity for cholinesterase inhibitory activity [64]. However, there are some exceptions to the aforementioned observation of structure–activity relationship in the literature. For example, acacetin-7-*O*-beta-*D*-galactopyranoside showed a stronger inhibitory activity than luteolin with an IC_50_ of 6.7 µM [67].

Compound **5** has been identified as a critical pharmacophore for AD treatment. Numerous studies have reported cinnamic acid derivative hybrids with quinoline [68], tryptamine [69], tertiary amine [70], tacrine [71], memantine [72], donepezil [73], and rivastigmine [74]. A tacrine and N-benzyl pyridinium hybrid with cinnamic acid exhibited a considerable improvement in AChE and BchE inhibition at the nanomolar level [71,75]. In contrast, a rivastigmine–hydroxycinnamic acid hybrid was shown to improve the BChE inhibitory potential only compared to rivastigmine and donepezil standards [74]. Omifoate A, a cinnamic acid ester derivative derived from *Pycnanthus angolensis*, demonstrated a greater inhibitory capacity than cinnamic acid, with an IC_50_ of 6.51 µg/mL for AChE and 9.07 µg/mL for BChE [76].

Compounds **3** and **4** exhibited moderate activity against AChE based on their IC_50_ values in Table 4. Previous in vitro studies revealed better AChE inhibitory activities for compounds **3** and **4**, individually [77,78]. The IC_50_ value for **3** was 55 μg/mL, found in [78], which was equivalent to our observation for β-sitosterol standard (Acros Organics, New Jersey, NJ, USA) (Table 4). Furthermore, the IC_50_ of **4** was reported to be 63 g/mL [77]. Despite their individual significant inhibitory effects against AChE, the IC_50_ values of **3** and **4** as a mixture exhibited decreased AChE inhibition potentials, with an IC_50_ of 78.44 ± 0.70 μg/mL, showing the potential antagonism impact of the **3** and **4** when tested together.

### 2.5. In Silico Cholinesterase Activity of Isolated Compounds

**Figure 3:**
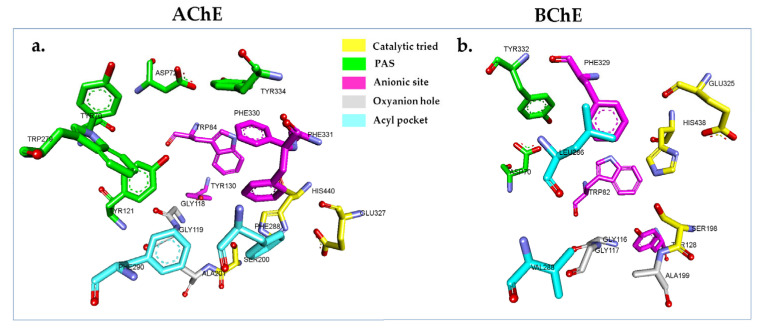
Binding site residues using BIOVIA Discovery Studio Visualizer (San Diego, CA, USA, 2019). (**a**). *Torpedo californica* acetylcholinesterase (*Tc*AChE) (PDB:1W6R). (**b**). *Homo sapiens* butyrylcholinesterase (*Hs*BChE) (PDB: 4BDS).

By performing docking simulations, reliable conformations of a ligand within an enzyme’s active site are attained to gain functional and structural insight into the mechanism of inhibition [79]. In this work, the relevant crystal structures of AChE and BChE complexes with the inhibitors were obtained from the protein data bank (PDB) database (https://www.rcsb.org, accessed on 1 September 2021) [80] to determine the binding affinity of the isolated compounds (**1**–**7**) with AChE and BChE. It is critical to highlight that the chosen crystal structure has to be compatible with the in vitro system, not mutated, and not covalently attached to an inhibitor. Additionally, a ligand-bound enzyme would be an ideal option for docking studies. In this study, *Electrophorus electricus* AChE (*Ee*AChE) was employed for the in vitro assay. Three crystal structures of *Ee*AChE with a resolution greater than 4 Å were present in the PDB database (PDB IDs: 1C2B, 1EEA, and 1C2O); however, resolutions at these levels are insufficient to provide accurate information about the binding site’s topography [81,82]. *Tetronarce californica* (*Tc*AChE, basionym: *Torpedo californica*) and *Ee*AChE share over 60% of sequence identity, and their active center gorges differ only by a PHE 330/TYR 337 mutation (*Tc*AChE/*Ee*AChE) [83,84]. Thus, the crystal structure of *Tc*AChE (PDB ID: 1W6R) in complex with galantamine (inhibitor) at a resolution of 2.05 Å was used instead of the *Ee*AChE crystal structure [85]. Similarly, homo sapiens BChE (*Hs*BChE, PDB ID: 4BDS) was used rather than equine serum BChE (*Eq*BChE), which was used in the in vitro assays due to the lack of a 3D structure of *Eq*BChE in the PDB database. In addition, *Eq*BChE and *Hs*BChE share ~90% of amino acid sequence identity, and the active site is conserved in both enzymes [86]. Hence, *Tc*AChE (1W6R.PDB) and *Hs*BChE (4BDS.PDB) with their inhibitors were utilized for docking to aid our understanding of their bindings [27]. The binding sites of *Tc*AChE and *Hs*BChE are narrow gorge that are located at the bottom of the enzymes at a depth ~20 Å [27,79]. The gorge binding site is composed of five important regions to facilitate the hydrolysis of ACh. These regions are the catalytic triad, anionic site, peripheral anionic site (PAS), oxyanion hole, and acyl pocket (Figure 3) [27,87].

The molecular docking study was initiated by redocking the co-crystallized inhibitors galantamine derivative and tacrine (controls docking) into the crystal structures of *Tc*AChE (PDB ID: 1W6R) and *Hs*BChE (PDB ID: 4BDS), respectively. Control docking demonstrated that the native ligands were capable of recreating their binding location with a root mean square deviation (RMSD) of 0.72 Å for the galantamine derivative and 1.31 Å for tacrine (Figure S9). RMSD values of <2.0 Å are generally considered acceptable [87,88,89,90,91,92]. Thus, the docking parameters used for control docking were used for molecular docking studies with compounds (**1**–**7**) and the galantamine standard. The free binding energy (F.B.E) and inhibition constant (Ki) of all docked compounds are summarized in Table 5.

The results showed that the galantamine derivative was docked into the *Tc*AChE binding site with good affinity (−8.71 kcal/mol). The inhibitor formed two H-bonds: one with the catalytic triad (SER200 at a distance of 2.19 Å) and one with the oxyanion hole site (GLY118 at a distance of 2.58 Å) (Figure S9a,b). The derivative also generated hydrophobic interactions with the anionic site residues TRP84, PHE330, and PHE331, as well as with the acyl pocket site residues PHE288 and PHE290. The redocked structure (galantamine derivative) also showed hydrophobic interaction with TRP233, a non-critical binding site residue located at the wall of the gorge binding site [93,94]. The findings also displayed that tacrine also entered the *Hs*BChE binding site and exhibited moderate binding affinity (−6.67 kcal/mol). The 2D molecular interaction demonstrated that tacrine was hydrophobically bound to the anionic site (TRP82) (Pi-Pi T-shaped and Pi-Alkyl). Furthermore, it also interacted with ALA328, a non-key binding site residue (Figure S9c,d).

Compound **1** has a positive free binding energy for both enzymes, indicating that it could not bind to the active site due to the fact that it is a straight long alkyl chain with a huge size that hampered its entry into the binding site. This result correlates with the in vitro study where the IC_50_ of **1** was greater than 150 µg/mL for BChE and 248.6 ± 2.24 µM for AChE. The docked compounds (**2**–**7**), galantamine, and the docking controls (galantamine derivative and tacrine) were superimposed into the binding site of *Tc*AChE and *Hs*BChE. It is worth noting here that the selected ligands were able to enter the binding site and formed a similar pose as the controls (Figure S10). Table 5 shows that the binding affinity of **3** and **4** were stronger (more negative; <−2 kcal/mol) towards the binding to *Tc*AChE and *Hs*BChE than galantamine derivative and tacrine, the co-crystallized ligands. This was followed by compound **2**, with a comparable binding affinity to the co-crystallized ligands (within the standard error deviation). The docking scores also showed that the **5**, **6**, and **7** bound to the *Tc*AChE and *Hs*BChE binding sites with lower affinity (less negative) than the controls. However, in vitro assays demonstrated that **5** and **6** had good inhibitory activities, which was supported by literature data.

**Figure 4:**
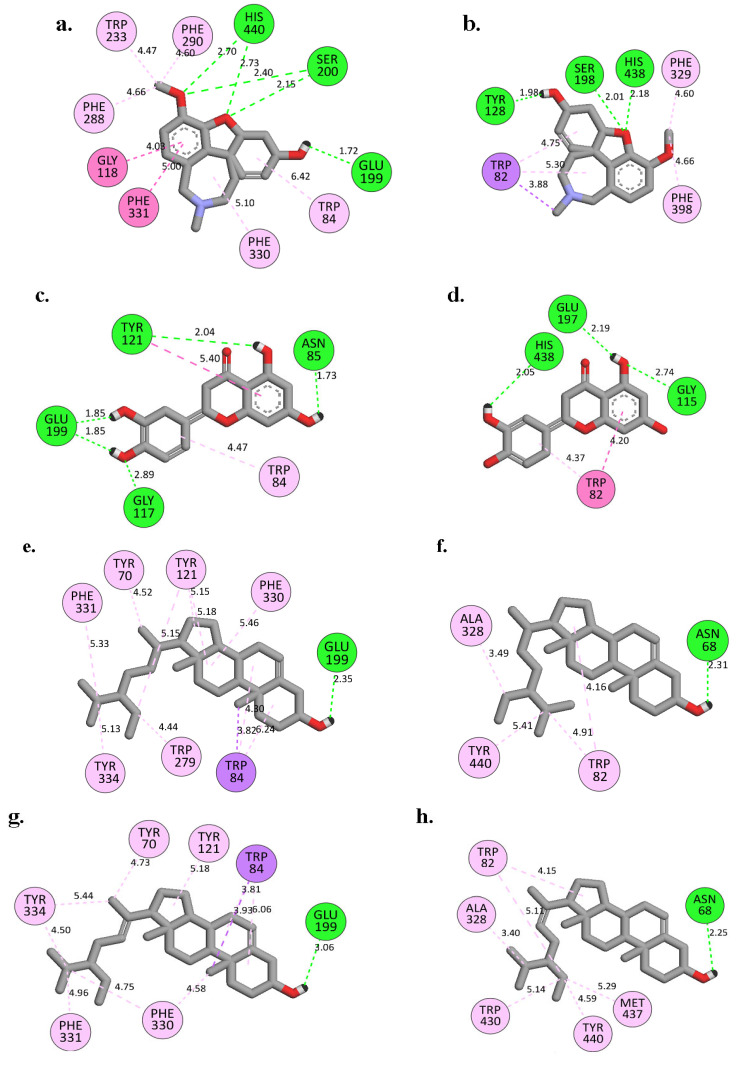

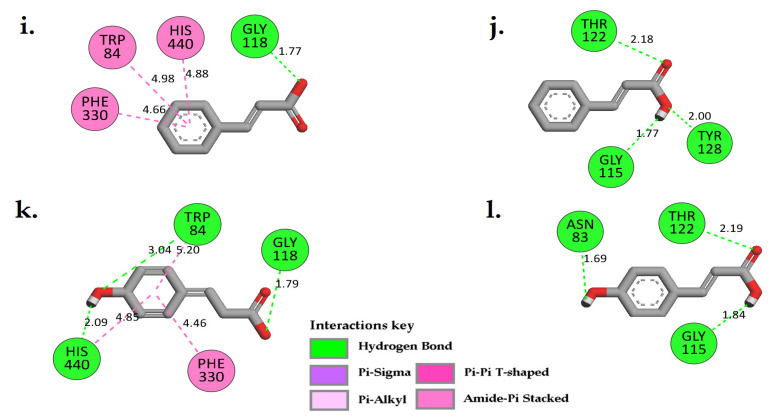
Two-dimensional interaction analysis for the docked complex towards TcAChE: (**a**) galantamine, (**c**) **2**, (**e**) **3**, (**g**) **4**, (**i**) **5**, and (**k**) **6**; and HsBChE (**b**) galantamine, (**d**) **2**, (**f**) **3**, (**h**) **4**, (**j**) **5**, and (**l**) **6** enzymes using BIOVIA Discovery Studio Visualizer.

Figure 4 and Table S1 display the molecular binding interactions analysis for the compounds (**2–6**) and the galantamine into the binding site of the enzymes (*Tc*AChE and *Hs*BChE). Galantamine bound to the active site pockets of the *Tc*AChE and *Hs*BChE with free binding energies of −9.63 and −8.38 kcal/mol, respectively. For *Tc*AChE (Figure 4a and Table S1), the oxygen atom from the methoxy group and the oxygen atom of the tetrahydrofuran ring of galantamine were involved in hydrogen bonding with both residues of the catalytic triad (SER200 and HIS440). Pi-Alkyl- and Pi-Pi T-shaped interactions (hydrophobic interactions) with the anionic sites (PHE330 and PHE331) were also observed. Additionally, the double bond of cyclohexene (C1=C2) of the galantamine faced toward the indole ring of TRP84 and stacked against the pi system of TRP84, forming a favorable hydrophobic interaction [27]. In addition, galantamine also aligned in a planar position and was stabilized via a hydrophobic interaction with the oxyanion hole (GLY118) and acyl pocket residues (PHE288 and PHE290) [27]. However, it is important to note that although GLU199 and TRP233 of *Tc*AChE participated in the interactions, they were not located within the main binding sites. A strong hydrogen bond between the hydroxyl oxygen of galantamine to the GLU199 was observed at a distance of 1.72 Å and hydrophobic interaction with TRP233. It was also observed that the oxygen atom of galantamine’s tetrahydrofuran ring created H-bonds with the catalytic tried residues (SER198 and HIS438) of the *Hs*BChE binding site (Figure 4b). TRP82 and PHE329 (anionic site) formed hydrophobic interactions with the cyclohexene and cycloheptane rings, as well as with the C17 atom of galantamine.

Compound **2** formed five H-bonds into the binding site of *Tc*AChE: one bond with TYR121 (PAS region, 2.04 Å) and four with the non-binding site residues, ASN85 (1.73 Å), GLY117 (2.89 Å), and GLU199 (1.85 Å). In addition, hydrophobic interactions were also observed to form with the anionic site and PAS (with TRP84 and TYR121, respectively) (Figure 4c). In *Hs*BChE, compound **2** formed three H-bonds with the binding site residues: one with the one of the catalytic triad residues, i.e., HIS438 (at distance 2.05 Å), and two H-bonds with GLY115 and GLU197 at distances of 2.74 Å and 2.19 Å, respectively (Figure 4d). Compound **2**’s hydrophobic interactions were mediated by tryptophan residues. TRP84 was found to be involved in the Pi-Alkyl interaction in *Tc*AChE, whilst, in *Hs*BChE, TRP82 was involved in Pi-Pi T-shaped interaction (Figure 4c,d). In vitro studies showed that compound **2** had the highest inhibition activity among all the isolated compounds towards AChE and BChE and was close to the control (galantamine). The strong activity might have been due to the fact that compound **2** formed multiple interactions, especially with TRP84 in the *Tc*AChE binding site and with the catalytic triad and anionic site residues in the *Hs*BChE.

Compounds **3** and **4** displayed similar binding poses in the *Tc*AChE and *Hs*BChE binding sites. In *Tc*AChE, **3** and **4** (Figure 4e,g) bound to the anionic site (TRP84, PHE330, and PHE331) and PAS (TYR70, TYR121, TYR334, and/or TRP279) via hydrophobic interactions. Compounds **3** and **4** formed one H-bond with GLU199 (2.35 Å with **3** and 3.06 Å with **4,** respectively). The **3** and **4** interactions with *Hs*BChE were limited to the anionic site, which was mediated by a hydrophobic interaction with TRP82. A strong H-bond was formed between ASN68 with **3** (2.31 Å) and **4** (2.25 Å), and hydrophobic interactions with non-main binding site residues such as TYR440, ALA328, and TRP430 were also detected (Figure 4f,h). However, the inhibitory effects of **3** and **4** were found to be moderate in the in vitro assay compared to galantamine, which could be due to the lack of interaction with the catalytic triad residues, as observed in galantamine and its derivative. A striking observation with regard to β-sitosterol (**3**) and stigmasterol (**4**) is that they had similar free binding energy and bound to the same amino acid in the binding site, implying that their presence as a mixture will negatively affect the binding into the *Tc*AChE and *Hs*BChE by competing at the same binding site. This could explain why there was a weaker inhibitory effect as a mixture in the in vitro assay.

Moreover, **5** and **6** bound in the same binding pose as **3** and **4** to the *Tc*AChE and *Hs*BChE binding sites. Compound **5** showed a strong H-bond with the oxyanion hole (GLY118) at a distance of 1.77 Å and with hydrophobic interactions with a catalytic triad (HIS440) and the anionic site (TRP84 and PHE330) in the *Tc*AChE binding site (Figure 4i). Furthermore, three H-bonds were also noted to form by this compound with the HsBChE: one in the anionic site at a distance of 2.0 Å and two with non-main binding site residues, i.e., with GLY115 (1.77 Å) and THR122 (2.18 Å) (Figure 4j). Compound **6** formed three H-bonds: one with the catalytic triad (HIS440, 2.09 Å), one with TRP84 at a distance of 3.04 Å (anionic site), and the third with GLY118 (oxyanion hole) at a distance of 1.79 Å. Furthermore, compound **6**’s aromatic ring generated hydrophobic interactions with the catalytic triad residue, HIS440, and anionic site (TRP84 and PHE330) of the *Tc*AChE (Figure 4k). Likewise, **6** formed three strong H-bonds with the non-main binding site of *Hs*BChE, including ASN83 (1.69 Å), GLY115 (1.84 Å), and THR122 (2.19 Å) (Figure 4l). These observations may help to explain the significant inhibitory effects of compounds **5** and **6** on AChE compared to BchE in the in vitro assays.

## 3. Materials and Methods

### 3.1. Materials and Instruments

Acetylcholinesterase (AchE) from *Electrophorus electricus* (electrical eels, type VI-S, 200–1000 unit/mg), Butyrylcholinesterase from equine serum (lyophilized powder ≥ 500 units/mg protein), substrate acetylthiocholine iodide (ATCI), S-butyrylthiocholine iodide (BTCI), sodium phosphate monobasic, and sodium phosphate dibasic were purchased from Sigma-Aldrich (St. Louis, MO, USA). The coloring agent 5,5-dithio-bis-[2-nitrobenzoic acid] (DTNB), and β-sitosterol were obtained from Acros Organics (Acros Organics, NJ, USA). Galantamine hydrobromide was obtained from Calbiochem (San Diego, CA, USA). Silica gel (Kieselgel 60, 230–400 mesh atm) was purchased from Merck (Darmstadt, Germany). The solvents were of analytical grade and used as received.

NMR data were collected using either a Bruker Biospin spectrometer ((Bruker BioSpin GmbH, Karlsruhe, Germany) at 500 MHz for ^1^H and 125 MHz for ^13^C or a Bruker advance III HD 700 MHz NMR spectrometer (Bruker BioSpin GmbH, Karlsruhe, Germany), equipped with a 5 mm BBO probe, operating at 700 MHz for ^1^H and 175 MHz for ^13^C. Residual solvent signals were applied for referencing. MS data were acquired using Agilent G6540B Accurate-Mass Q-TOF LC/MS (Agilent Technologies, Santa Clara, CA, USA). The analytes were converted and fragmented into charged ions by the Dual AJS ESI and were brought into the mass spectrometer (LC-QTOF-MS/MS system) (Agilent Technologies, Santa Clara, CA, USA). A negative ion mode mass spectrum was generated. MS data were also obtained using GC (HP 6890 series GC system, Hewlett-Packard, Palo Alto, CA, USA), equipped with an autosampler (HP 7683 series injector) and coupled with a mass selective detector (HP 5973) using a cross-linked 5% phenylmethylsiloxane capillary column (30 m × 0.25 mm i.d., 0.25 film thickness).

### 3.2. Plant Materials 

Flowers of *C. timoriensis* were collected during the flowering stage in June–August 2019 from Universiti Sains Malaysia-main campus, while the pods of *C. grandis* were collected in January 2020 from Kedah, Malaysia. The raw materials of both plants were identified by a plant taxonomist, Dr. Rahmad Zakaria, and the voucher specimen (11852) for *C. timoriensis* and (11851) for *C. grandis* were deposited in the herbarium of the School of Biological Sciences, USM, Penang, Malaysia. The collected plant materials were air-dried in the shade away from direct sunlight. The dried plant materials were ground into coarse powder and stored at room temperature, protected from light, until required for further analysis.

### 3.3. Plant Preparation and Extraction

*C. grandis* pods (1.5 kg) were cleaned of dust, and the seeds were removed. Then, they were extracted using different solvents with increasing polarities via soxhlet apparatus. First, 500 mL of *n*-hexane was placed in the distillation round bottom flask and connected to the main chamber, which contained the plant materials, and attached to the condenser. The extraction procedure was continued until the solvent in the soxhlet solvent arm turned colorless. Then, *n*-hexane used was collected and evaporated under pressure at 40 °C. The dried residue was collected and preserved in a clean vial, namely the hexane fraction (1.84 g). The previous extraction process was repeated with a higher-polarity solvent, ethyl acetate, using the same plant material to obtain the ethyl acetate fraction (23.96 g). This was followed by methanol, which provided the methanol fraction (168.57 g). Finally, distilled water was used to obtain the aqueous extract (24.97 g). The dried flowers of *C.* *timoriensis* (300 g) were cleaned and extracted using the same described method via soxhlet apparatus to yield four different extracts, namely *n*-hexane (11.5 g), ethyl acetate (43.12 g), methanol (35.38 g), and aqueous (30.08 g) extracts. To achieve the maximum yield, the previous extraction procedure was repeated three times using fresh solvent every time. The extraction process was continued until the solvent turned colorless in the soxhlet solvent arm.

### 3.4. LC-MS/MS Analyses of C. timoriensis and C. grandis Extracts 

Separation was performed using a Thermo Scientific C18 column (Termo Fisher Scientifc, Waltham, MA, USA, AcclaimTM Polar Advantage II, 3 × 150 mm, 3 μm particle size) on an UltiMate 3000 UHPLC system (Dionex, Idstein, Germany). Gradient elution was performed at a flow rate of 0.4 mL/min at a 40 °C column temperature using H_2_O + 0.1% Formic Acid (A) and 100% ACN (B) with a 22 min total run time. The injection volume of the sample was 3 µL. The gradient started at 5% B (0–3 min); 80% B (3–10 min); 80% B (10–15 min); and 5% B (15–22 min). High-resolution mass spectrometry was carried out using a MicroTOF QIII Bruker Daltonic ((Bruker Daltonik GmbH, Bremen, Germany) using ESI-negative ionization with the following settings: capillary voltage: 4000 V; nebulizer pressure: 2.0 bar; drying gas: 8 L/min at 300 °C. The mass range was at 50–1000 *m/z*. The accurate mass data of the molecular ions, provided by the TOF analyzer, were processed using Compass Data Analysis software, version 4.2 (Bruker Daltonik GmbH, Bermen, Germany).

### 3.5. General Isolation Procedure

The *n*-hexane extract of *C. timoriensis* flowers (3.00 g) was subjected to column chromatography (silica gel 60, 230–400 ASTM) to yield 25 fractions. The fractions were eluted using a gradient mobile phase starting from 100% *n*-hexane until 100% ethyl acetate was reached. The purification of fraction no. 11 (120 mg, 20% ethyl acetate: 80% *n*-hexane) was carried out using small purification normal column chromatography with an isocratic elution of (10% ethyl acetate: 90% *n*-hexane) to yield compound **1** (46.2 mg) (Figure S1). 

On the other hand, ethyl acetate extract of *C. timoriensis* (40 g) was subjected to vacuum liquid chromatography (VLC) to separate the compounds into several fractions based on their polarity. The VLC system consisted of a VLC chamber in which the adsorbent (Silica gel 60 PF_254_, Merck, Darmstadt, Germany) was placed, a vacuum pump, and a stoppered Erlenmeyer flask to collect the eluent. The elution of VLC was started with 100% hexane moving to 100% ethyl acetate, and the polarity was increased until 100% methanol was reached. Thirteen fractions were obtained after TLC profiling, and the pooling of similar fractions was carried out.

VLC fraction no. 2 (6.00 g, 10% ethyl acetate: 90% *n*-hexane) was subjected to a normal silica gel 60 (mesh 230–400 ASTM) column and eluted with a mixture of solvents of increasing polarity of *n*-hexane: ethyl acetate (100% hexane up to 100% ethyl acetate (*v*/*v*)). The eluted sub-fractions were monitored on TLC (silica gel 60 F_254_, Merck, Darmstadt, Germany) using *n*-hexane: ethyl acetate mobile phase system or chloroform: methanol. Sub-fractions with similar TLC profiles were combined and pooled together to give 30 sub-fractions. The purification of sub-fraction 20 (190 mg, eluted with 40% ethyl acetate and 60% *n*-hexane) provided 21.5 mg of a yellow powder, compound **2**. The purification was accomplished through preparative thin layer chromatography with 15% methanol: 85% chloroform as the mobile phase.

Moreover, ethyl acetate extract (6.5 g) of *C. grandis* was purified using normal silica column chromatography (silica gel 60, 230–400 mesh ASTM). The elution was carried out with a stepwise gradient solvent system using *n*-hexane: ethyl acetate, affording 28 major fractions. Fraction 8 (15% ethyl acetate) was subjected to a solvent purification step to yield 51.8 mg of a mixture of compounds **3** and **4**. Fraction 15 (25% ethyl acetate, 1.95 g) was further purified using small purification column chromatography. Gradient elution was carried out using a 100% chloroform to 100% methanol system to yield 20 sub-fractions; sub-fraction 6 yielded compound **5** (10% methanol, 398 mg), and sub-fraction 13 (30% methanol, 16.1 mg) provided compound **6** (Figure S1). Methanol extract of *C. grandis* (100 g) was subjected to an acid hydrolysis (2N-hydrochloric acid and heated to reflux at 80 °C for 60 to 90 min) step to separate the glycoside part and enhance the isolation process using normal column chromatography. The resulting hydrolyzed fraction (10.5 g) was subjected to silica gel column chromatography with a gradient mobile phase of 100% *n*-hexane to 100% ethyl acetate to yield 35 fractions. The purification of fraction 14 (25% ethyl acetate) yielded a 52.6 mg of mixture of compounds **3** and **4**. Finally, fraction 22 (2.95 g, 40% ethyl acetate) was also subjected to purification column with a gradient mobile phase of 100% chloroform to 100% methanol to give compound **7** (90.1 mg), which was eluted at 10% methanol.

(**1**). Arachidyl arachidate 

White powder; GC-MS *m*/*z* 592.7 [M]^+^ (calculated for C_40_H_80_O_2_: 592.62];^1^H NMR (500 MHz, CDCl_3_): δ 4.05 (t, *J* = 6.6 Hz, 2H), 2.29 (t, *J* = 7.5 Hz, 2H), 1.61, 1.27, 0.88 (t, *J* = 7.0 Hz, 6H).^13^C NMR (125 MHz, CDCl_3_): δ 174.1, 64.4, 34.4, 31.9, 29.7 (2), 29.6 (2), 29.5 (2), 29.4, 29.3 (3) 28.7, 26.0, 25.1, 22.7, 14.1.

(**2**). Luteolin 

Light-yellow powder; LC-MS *m*/*z* 285.0401 [M-H]^−^ (calculated for C_15_H_10_O_6_: 286.24); ^1^H NMR (700 MHz, CD_3_OD) δ 7.86, 7.85 (d, *J* = 8.3 Hz, 1H), 7.39 (d, *J* = 8.3 Hz, 1H), 6.99, 6.92, 6.70. ^13^C NMR (175 MHz, CD_3_OD) δ 184.0, 166.5, 166.0, 163.3, 159.5, 150.9, 147.1, 124.1, 120.4, 116.9, 114.5, 105.5, 104.2, 100.3, 95.1. 

β-sitosterol (**3**) and Stigmasterol (**4**)

While needle crystals; GC-MS *m*/*z* 414 [M]^+^ (calculated for C_29_H_50_O: 414.71); *m*/*z* 412 [M]^+^ (calculated for C_29_H_48_O: 412.69); ^1^H NMR (500 MHz, CDCl_3_): δ 5.35, 5.14 (dd, *J* = 15.1, 8.7 Hz, 1H), 5.02 (dd, *J* = 15.1, 8.7 Hz, 1H), 3.53, 2.28, 2.00, 1.84, 1.59, 1.01, 0.80, 0.68. ^13^C NMR (125 MHz, CDCl_3_): δ 140.9, 138.5, 129.4, 121.9, 72.0, 57.0, 56.9, 56.2, 56.1, 51.4, 50.3, 46.0, 42.5 (2), 42.4, 40.7, 39.9, 39.8, 37.4, 36.7, 36.3, 34.1, 32.1, 31.8, 29.3, 29.1, 28.4, 26.2, 25.6, 24.5 (2), 23.2, 21.4, 21.2, 20.0, 19.6, 19.2, 19.1, 18.9, 12.4, 12.2, 12.1, 12.0.

(**5**). Cinnamic acid

White amorphous crystals: LC-MS *m*/*z* 147.0449 [M-H]^−^ (calculated for C_9_H_8_O_2_: 148.1586)]; ^1^H NMR (700 MHz, CD_3_OD): δ 7.64 (d, *J* = 16.0 Hz, 1H), 7.54 (m, 2H), 7.36 (dd, *J* = 5.0, 2.0 Hz, 3H), 6.45 (d, *J* = 16.0 Hz, 1H). ^13^C NMR (175 MHz, CD_3_OD): δ 170.3, 146.3, 135.8, 131.4, 130.0, 129.2, 119.3.

(**6**). 4-Hydroxycinnamic acid

Light-yellow amorphous crystals; GC-MS *m*/*z* 164.9 [M]^+^ (calculated for C_9_H_8_O_3_: 164.0473); ^1^H NMR (500 MHz, CD_3_OD): δ 7.59 (d, *J* = 15.9 Hz, 1H), 7.43(d, *J* = 8.7 Hz, 2H), 6.79 (d, *J* = 8.7 Hz, 2H), 6.27 (d, *J* = 15.9 Hz, 1H). ^13^C NMR (125 MHz, CD_3_OD): δ 171.4, 161.2, 146.6, 131.1, 127.3, 117.2, 115.6.

(**7**). 4-Hydroxymethylfurfural

White needle crystals: GC-Ms [*m/z* 126.0 [M]^+^ (calculated for C_6_H_6_O_3_: 126.11)]; ^1^H NMR (500 MHz, CDCl_3_): δ 9.61, 7.21 (d, *J* = 3.6 Hz, 1H), 6.56 (d, *J* = 3.5 Hz, 1H), 4.62 (s, 2H). ^13^C NMR (125 MHz, CDCl_3_): δ 177.8, 157.3, 152.9, 122.0, 112.0, 64.7.

### 3.6. In Vitro Cholinesterase Assay

The anti-cholinesterase activity of plant extracts, isolated compounds (**1**–**7**), and standards (galantamine and β-sitosterol) were determined in triplicate using Ellman’s method described previously [58]. A series of dilutions of plant extracts (3.125 ppm to 200 ppm), pure compounds (1.5625 ppm to 100 ppm), and galantamine standard (1.5625 ppm to 50 ppm) were freshly prepared in dimethyl sulfoxide (DMSO). Using a 96-well plate, 179 µL of 0.05 mM sodium phosphate buffer (pH 7.5), 1 µL of sample, and 10 µL of 0.5 unit/mL AChE enzyme were added. Then, after 15 min of incubation at 25 °C, 5 µL of 10 mM DTNB and 5 µL 14 mM ATCI were added to give a final reaction volume of 200 µL with a final DMSO ratio of 0.05%. After 30 min of incubation, the absorption was measured at 415 nm using a Promega Glomax^®^ Multi Plus Reader (Promega, Sunnyvale, CA, USA). Each run was carried out in triplicate on three consecutive days. Anti-BChE assays were performed using the same procedures described above but using BChE from equine serum (lyophilized powder ≥ 500 units/mg protein) instead of AChE and BTCI instead of ACTI. The final absorbance of each tested sample was calculated by subtracting the absorbance of their respective blank without enzyme addition. The enzymes’ inhibition values were calculated using Equation (1). Then, the IC_50_ values of all tested samples and standards were calculated from the curve of the % inhibition versus concentration.

(1) $$\%\;\mathrm{Inhibition}=\frac{\mathrm{Abs}\;\left(-\right)\mathrm{ve}\;\mathrm{control}-\mathrm{Abs}\;\mathrm{test}\;\mathrm{sample}}{\mathrm{Abs}\;\left(-\right)\mathrm{ve}\;\mathrm{control}}\times 100$$

### 3.7. In Silico Molecular Docking

The X-ray crystal structures of the *Tc*AChE (*Torpedo californica*, PDB ID: 1W6R) and *Hs*BChE (*Homo sapiens*, PDB ID: 4BDS) enzymes’ complexes with the inhibitors, galantamine derivative and tacrine, respectively, were retrieved from the RCSB Protein Data Bank (PDB) (https://www.rcsb.org, accessed on 1 September 2021) [81,86,95]. All water and heteroatoms (except the inhibitors) were deleted from the structures using Biovia Discovery Studio Visualizer (San Diego, CA, USA, 2019) [95]. To prepare the molecular system (enzymes) for the molecular docking process, the PDB2PQR web service (https://pdb2pqr.poissonboltzmann.org/pdb2pqr, accessed on 13 August 2021) was used to perform additional calculations on the enzymes, such as reconstructing missing atoms, assigning atomic charges, and radii using the SWANSON force field (AMBER ff99 charges with optimized radii) [91,96]. The protein was exposed to the most widely used empirical pKa predictor (PROPKA3), which was set to pH 7.00, to assign the protonation states of the ionizable groups [97]. Finally, the enzymes were submitted to the MolProbity web service (http://molprobity.biochem.duke.edu/, accessed on 13 August 2021) for contact atoms’ corrections and to add the missing hydrogen atoms [91,97].

In this study, the compounds (**1**–**7**) isolated from *C. timoriensis* and *C. grandis* were sketched using PerkinElmer ChemDraw Professional 17.1 (PerkinElmer, Massachusetts, USA) [91] and used as ligands in the docking process. The ligands were subjected to energy minimization using the Molecular Mechanics 2 (MM2) force field by PerkinElmer Chem3D 17.1 (PerkinElmer, Waltham, MA, USA) [91]. Then, the minimized structures (ligands) were saved in a PDB format. The inhibitors (galantamine derivatives and tacrine) were used as controls in the docking process for comparison purposes with the obtained ligands.

AutoDock Tools 1.5.6 (The Scripps Research Institute, San Diego, CA, USA) was used for the preparation of enzymes and ligands for the docking simulation step [98]. For the enzymes, polar hydrogen and Kollman charges were added to it, while Gasteiger charges were assigned for the inhibitors and ligands. The charged structures were saved in PDBQT format. The grid box was centered on the binding site of the enzymes. The coordinates of the *Tc*AChE were set at center x = 3.518, y = 65.122, and z = 64.481 [27] and x =133.076, y = 116.113, and z = 41.335 for *Hs*BChE. The size of the grid box was 40 × 40 × 40 (x, y, and z) with a spacing of 0.375 for the enzymes. AutoDock 4.2 was used to simulate the docking process [99], where the enzymes were set as rigid and the ligands as flexible. The number of docking runs was set at 150, the population size was 150, the maximum number of evals was 2,500,000 (medium), and the maximum number of generations was 27,000. The Lamarckian genetic algorithm was chosen to perform this process, and the remaining parameters were kept as default and saved in the docking parameter files (DPFs). Molecular interactions between the ligands and the enzymes were analyzed and visualized (2D and 3D) using BIOVIA Discovery Studio Visualizer 19 (San Diego, CA, USA, 2019) [95].

## 4. Conclusions

In this study, the anti-cholinesterase and chemical investigation of *C. timoriensis* and *C. grandis* resulted in the isolation and identification of seven compounds. To the best of our knowledge, all these compounds except **3** were isolated from these plant species for the first time. Compound **2** was found to be active toward AChE and BChE (IC_50_ of 20.47 ± 1.10 and 46.15 ± 2.20 µM), followed by **5** and **6**, which showed comparable activity with an IC_50_ of 40.5 ± 1.28 and 43.4 ± 0.61 µM, respectively, against AChE. However, **5** and **6** exhibited moderate activity toward BChE with IC_50_ values of 373.1 ± 16.4 and 409.17 ± 14.80 µM, whereas **3** and **4** demonstrated slightly moderate activity as a mixture. The molecular docking simulations were performed to understand the inhibitory mechanism of the identified compounds. In general, the calculated binding energies were in agreement with the IC_50_ values obtained from the in vitro studies. Strong hydrogen bonding and hydrophobic interactions with residues at the PAS and anionic sites of the binding cavity might possibly contribute to the low IC_50_ values of **2**, **5**, and **6** toward AChE and BChE.

## Figures and Tables

**Table 1:** The inhibitory activity of *C. grandis* extracts against acetylcholinesterase enzyme.

Sample	% Inhibition *	IC_50_ (µg/mL)
Galantamine	100.36% ± 0.49	1.40 ± 0.12
Aq- extract	51.23% ± 0.07	193.83± 0.19
MeOH extract	83.90% ± 0.75	74.09 ± 1.60
EA extract	86.19% ± 1.75	82.96 ± 2.81
*n*-hex extract	47.70% ± 0.35	222.44 ± 4.30

* Data are presented as mean ± SD (*n* = 3); * % inhibition at 200 µg/mL.

**Table 2:** Liquid chromatography–tandem mass spectrometry (LC–MS/MS) of ethanolic extract *Cassia timoriensis*.

Peak No.	Rt (min)	(M-H)^−^	Molecular Weight	Error	Molecular Formula	LC-MS/MS Fragmentation	Predicted Compounds
3	1.7	131.0467	132.0422	−6.35	C_5_H_8_O_4_	-	Methyl succinic acid
7	1.8	341.1114	342.1169	4.39	C_19_H_18_O_6_	179.0566 (100%)	Lactose
8	1.8	387.1146	388.1217	4.39	C_13_H_24_O_13_	113.0247, 19.0346, 149.0456, 179.0559, 341.1095 (100%)	2,3,4,5,6-pentahydroxy-7-[(2S,3R,4S,5S,6R)-3,4,5-trihydroxy-6-(hydroxymethyl)oxan-2-yl]oxyheptanoic acid
9	1.8	683.2288	684.2338	2.92	C_25_H_40_N_4_O_18_	161.0462, 179.0561, 341.1108 (100%)	2-*O*-[1-[4-(alpha-*D*-Mannopyranosyl)-1H-1,2,3-triazole-1-yl]-1,3-dideoxy-beta-*D*-galactopyranose-3-yl]-*N*-acetyl-alpha-neuraminic acid
10	1.8	401.1314	402.1373	2.74	C_14_H_26_O_13_	193.0728 (100%)	2-(hydroxymethyl)-3,4,5-trihydroxy-6-(hydroxymethyl)oxan-2-yl]peroxyethoxy]oxane-3,4,5-triol
11	1.8	179.0565	180.0633	1.11	C_6_H_12_O_6_	-	D-Mannose
16	2.3	179.0570	180.0633	3.90	C_6_H_12_O_6_	-	D-Galactose
17	2.3	191.0567	192.0633	2.09	C_7_H_12_O_6_	127.0410, 191.0579 (100%)	Quinic acid
23	7.7	441.1433	442.1481	4.53	C_20_H_26_O_11_	233.0476, 321.1000 (100%)	Obtusichromoneside A
23	7.7	397.1167	398.1218	1.75	C_18_H_22_O_10_	233.0483 (100%), 277.0738	Obtusichromoneside C
28	8.9	435.1316	436.1369	1.37	C_21_H_24_O_10_	151.0406 (100%), 313.0942	Catechin-3-rhamnoside
31	9.0	609.1496	610.1533	−0.49	C_27_H_30_O_16_	300.0296 (100%)	Rutin
33	9.1	447.0950	448.1000	3.11	C_21_H_20_O_11_	285.0418 (100%)	luteolin-7-*O*-glucoside
42	9.5	561.1428	562.1475	4.09	C_30_H_26_O_11_	271.0632 (100%), 255.5562	Epicatechin-(4beta-8)-epiafzelechin
43	9.6	833.2106	834.2159	2.04	C_45_H_38_O_16_	271.0630 (100%), 409.0955, 561.1431	ent-Fisetinidol-(4β→8)-catechin-(6→4β)-ent-fisetinidol
45	9.8	552.1378	553.1346	2.44	C_28_H_25_O_12_^+^	151.0403, 271.0634 (100%), 391.0835, 568.7994	Cyanidin 3-(6″-benzoyl)gulucoside
52	10	817.2154	818.2210	1.71	C_45_H_38_O_15_	255.0673, 271.0629 (100%),	6,8-bis [2-(3,4-dihydroxypheny)-3,7-dihydroxy-3,4-dihydro-2H-1-benzopyran-4-yl]-2-(3-hydroxyphenyl)-3,4-dihydro-2H-1-benzopyran-3,5,7-triol
56	10.3	817.2159	818.2140	3.90	C_45_H_38_O_15_	255.0673, 271.0628 (100%), 409.0949	[Epiafzelechin-(4β→8)]2-epiafzelechin
62/63	10.6	301.0360	302.0427	0.99	C_15_H_10_O_7_	149.0251, 301.0367(100%)	Quercetin
71	11.4	285.0416	286.0477	3.15	C_15_H_10_O_6_	133.030, 285.0419 (100%),	Luteolin
72	11.4	285.0416	286.0477	1.32	C_15_H_10_O_6_	133.030, 285.0419 (100%)	Kaempferol
73	11.5	571.0894 [2M-H]^−^	286.0477 [572.0955]	1.57	C_30_H_20_O_12_	285.0421 (100%), 298.4290, 504.3217	2″,3″-Dihydro-5′,6″-biluteolin
76	11.8	299.0539	300.0639	−2.78	C_16_H_12_O_6_	215.6827, 256.0391, 284.0351 (100%)	Chrysoeriol
76	11.8	299.0539	300.0633	5.79	C_16_H_12_O_6_	284.0351 (100%), 256.0391, 215.6276	Diosmetin

**Table 3:** Liquid chromatography–tandem mass spectrometry (LC–MS/MS) of ethanol extract *Cassia grandis*.

Peak No.	Rt (min)	*m/z* (M-H)^-^	Molecular Weight	Error	Molecular Formula	LC-MS/MS Fragmentation	Predicted Compounds
1	1.8	683.2264	684.2338	−0.58	C_25_H_40_N_4_O_18_	113.02, 19.03, 149.04, 79.05, 180.05, 341.11 (100%)	2-*O*-[1-[4-(alpha-*D*-Mannopyranosyl)-1H-1,2,3-triazole-1-yl]-1,3-dideoxy-beta-*D*-galactopyranose-3-yl]-*N*-acetyl-alpha-neuraminic acid
2	1.8	179.0555	180.0634	−5.02	C_6_H_12_O_6_	-	Mannose
3	1.8	341.1093	342.1162	0.29	C_12_H_22_O_11_	113.02, 179.05 (100%)	Lactose
3	1.8	341.1093	342.1162	0.29	C_12_H_22_O_11_	113.02, 19.03, 143.03, 147.25, 149.04, 161.04, 179.05 (100%)	Sucrose
4	2.0	377.0859	378.0951	−4.77	C_18_H_18_O_9_	179.05 (100%), 215.03	4-hydroxy-5-[(2S,3R,4S,5S,6R)-3,4,5-trihydroxy-6-(hydroxymethyl)oxan-2-yl]oxy-1H-benzo[f][2]benzofuran-3-one
5	2.0	387.1146	388.1217	−0.25	C_13_H_24_O_13_	113.02, 149.04, 179.05, 341.11 (100%)	2,3,4,5,6-pentahydroxy-7-[(2S,3R,4S,5S,6R)-3,4,5-trihydroxy-6-(hydroxymethyl)oxan-2-yl]oxyheptanoic acid
8	2.3	267.0720	268.0794	−1.49	C_9_H_16_O_9_	113.02, 116.15, 267.07 (100%)	2,3,4,5,6,8-hexahydroxy-9-oxononanoic acid
9	2.8	290.0886	291.0954	0.68	C_11_H_17_NO_8_	128.035(100%), 200.05	N-Fructosyl pyroglutamate
12	7.8	471.1718	472.1792	−0.84	C_18_H_32_O_14_	179.05, 341.10 (100%)	4,6-deoxy-*L*-xylHex(a1-2)[Gal(a1-3)]Gal
15	8.1	409.1730	410.1788	2.93	C_17_H_30_O_11_	113.02, 115.07, 205.07 (100%)	5-ethyl-3,4,6-trihydroxyoxan-2-yl]methoxymethyl]-4,5-dihydroxy-6-(methoxymethyl)oxane-2-carboxylic acid
17	8.3	539.1980	540.2054	−0.74	C_22_H_36_O_15_	205.06 (100%)	Ethyl 4-*O*-[3-*O*-(carboxymethyl)-beta-*D*-galactopyranosyl]-6-*O*-(alpha-*L*-fucopyranosyl)-2,3-dideoxy-beta-*D*-erythro-hexa-2-enopyranoside
20	8.7	553.2149	554.2211	1.44	C_23_H_38_O_15_	205.07(100%), 209.60, 247.08, 289.12	-4,5-dihydroxy-6-(hydroxymethyl)-3,4,5-trihydroxy-6-methyloxan-2-yl]oxycyclohexyl]oxyoxan-3-yl]oxyethyl]propanedioic acid
22	9.0	447.0932	448.1005	−0.67	C_21_H_20_O_11_	285.040(100%)	luteolin-7-*O*-glucoside
26/27	10.8	147.0450	148.0524	2.70	C_9_H_8_O_2_	-	Trans-cinnamic acid
32	11.8	293.1762	294.1831	0.34	C_17_H_26_O_4_	221.15 (100%), 236.10	6-(3-hydroxy-5-pentyl-phenoxy)hexanoic acid
30	11.4	285.0402	286.0477	−0.07	C_15_H_10_O_6_	133.02, 285.04 (100%)	kaempferol
44	14.0	279.2330	280.2402	−0.71	C_18_H_32_O_2_	279.23 (100%)	Linoleic acid

**Table 4:** The cholinesterase inhibitory potentials of the isolated compounds.

Compounds	AChE (IC_50_)		BChE (IC_50_)	
	µg/mL	µM	µg/mL	µM
**1**	147.45 ± 1.33	248.6 ± 2.24	>150	-
**2**	5.86 ± 0.31	20.47 ± 1.10	13.21 ± 0.63	46.15 ± 2.20
**3** and **4**	78.44 ± 0.70	-	87.29 ± 3.61	-
**5**	6.00 ± 0.19	40.5 ± 1.28	55.28 ± 2.44	373.1 ± 16.4
**6**	7.12 ± 0.10	43.4 ± 0.61	67.17 ± 2.43	409.17 ± 14.80
**7**	19.93 ± 0.44	158.04 ± 3.49	>100	-
β-sitosterol	56.84 ± 3.59	137.06 ± 8.66	64.98 ± 3.01	156.7 ± 7.26
Galantamine	1.40 ± 0.12	4.87 ± 0.42	3.73 ± 0.14	12.98 ± 0.49

**Table 5:** Free binding energy (F.B.E) and the inhibition constant (Ki) values of the inhibitors and the isolated compounds (**1–7**) towards the enzymes (*Tc*AChE and *Hs*BChE).

Compounds	*Tc*AChE		*Hs*BChE	
	F.B.E. (kcal/mol)	Ki (µM)	F.B.E. (kcal/mol)	Ki (µM)
**1**	+++ *	+++ *	+++ *	+++ *
**2**	−8.40	0.70	−7.21	5.19
**3**	−11.29	0.01	−9.92	0.05
**4**	−11.22	0.01	−10.00	0.05
**5**	−5.16	164.78	−4.98	222.07
**6**	−5.43	104.70	−5.36	116.93
**7**	−4.60	428.09	−4.48	522.47
Galantamine	−9.63	0.14	−8.38	0.72
Galantamine derivative	−8.71	0.41	-	-
Tacrine co-crystalized	-	-	−6.67	12.94

* +++: The reaction between the ligand and the enzyme is not spontaneous and is not favorable.

## Data Availability

Not applicable.

## Supplementary Materials

The following supporting information can be downloaded at: https://www.mdpi.com/article/10.3390/plants12020344/s1, Figure S1: The isolation scheme of *C. timoriensis* and *C. grandis*; Figure S2: Mass spectra of compounds **1**–**7**; Figure S3: ^1^H-NMR [CDCl_3_, 500 MHz] and ^13^C-NMR of [CDCl_3_, 125 MHz] of compound **1**; Figure S4: ^1^H-NMR [CDCl3, 700 MHz] and ^13^C-NMR of [CDCl3, 175 MHz] of **2**; Figure S5: ^1^H-NMR [CDCl_3_, 500 MHz] and ^13^C-NMR of [CDCl_3_, 125 MHz] of mixture of **3** and **4**; Figure S6: ^1^H-NMR [CDCl3, 700 MHz] and ^13^C-NMR of [CDCl3, 175 MHz] of **5**; Figure S7: ^1^H-NMR [CD_3_OD, 700 MHz] and ^13^C-NMR of [CD_3_OD, 175 MHz] of **6**; Figure S8: ^1^H-NMR [CDCL3, 500 MHz] and ^13^C-NMR of [CDCL3, 125 MHz] of **7**; Figure S9: Two-dimensional molecular interaction binding models, as well the superimposed co-crystallized structural pose (blue) with the docked structure (red) of *Tc*AChE-galantamine derivative (a,b) with RMSD = 0.72 Å. Additionally, (c,d) for *Hs*BChE-tacrine (RMSD = 1.32 Å); Figure S10: All docked compounds **1** (green), **2** (brown), **3** (orange), **4** (red), **5** (yellow), **6** (purple), **7** (gold), and the in vitro assay control (galantamine (gray)) were superimposed into the binding sites of *Tc*AChE (PDB ID: 1W6R) (a) and *Hs*BChE (PDB ID: 4BDS) (b). The co-crystalized ligands for *Tc*AChE (galantamine derivative) and *Hs*BChE (tacrine) are in blue color. Parts of the ribbon structure were removed to improve visualization; Table S1: Interaction analysis for galantamine and chosen compounds **2**–**6** after docking into *Tc*AChE and *Hs*BChE binding sites.
