# Supplementary material for: Potential Anti-Cholinesterase Activity of Bioactive Compounds Extracted from Cassia grandis L.f. and Cassia timoriensis DC
Source: Plants (Basel). 2023 Jan 11;12(2):344. doi: 10.3390/plants12020344 (PMC9862305; doi:10.3390/plants12020344)
Supplement: Supplementary file 1 [file plants-12-00344-s001.zip › plants-1799170-supplementary.pdf]

Supplementary Data

Potential Anti-Cholinesterase Activity of Bioactive Compounds extracted from *Cassia grandis* L.f. and *Cassia timoriensis* DC.

Maram B. Alhawarri [1,2], Roza Dianita [1], Mira Syahfriena Amir Rawa [1,3], Toshihiko Nogawa [3,4] and Habibah A. Wahab [1,3,*]

[1] School of Pharmaceutical Sciences, Universiti Sains Malaysia, Minden 11800, Malaysia
[2] Faculty of Pharmacy, Jadara University, Irbid 21110, Jordan
[3] USM-RIKEN Interdisciplinary Collaboration for Advanced Sciences (URICAS), Universiti Sains Malaysia, Gelugor 11800, Malaysia
[4] Molecular Structure Characterization Unit, Technology Platform Division, RIKEN Center for Sustainable Resource Science, 2-1 Hirosawa, Saitama 351-0198, Japan
* Correspondence: habibahw@usm.my; Tel.: +60-4-6577888

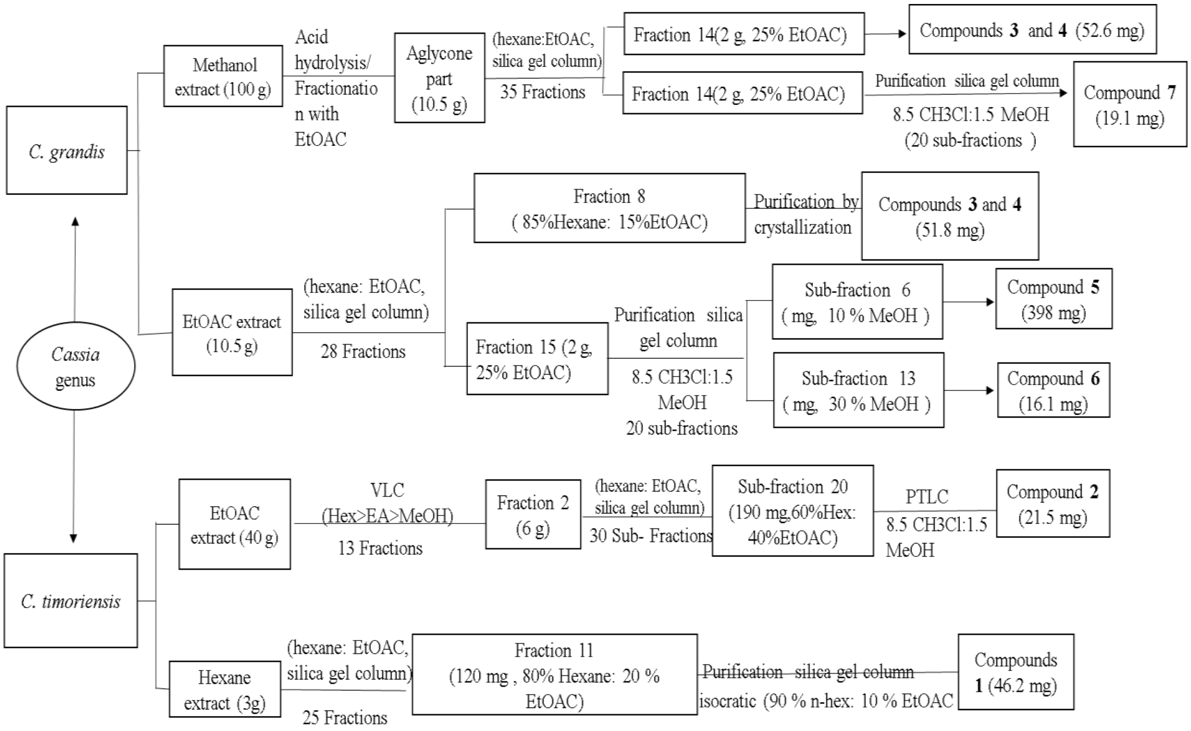

Figure S1. The Isolation scheme of *C. timoriensis* and *C. grandis*.

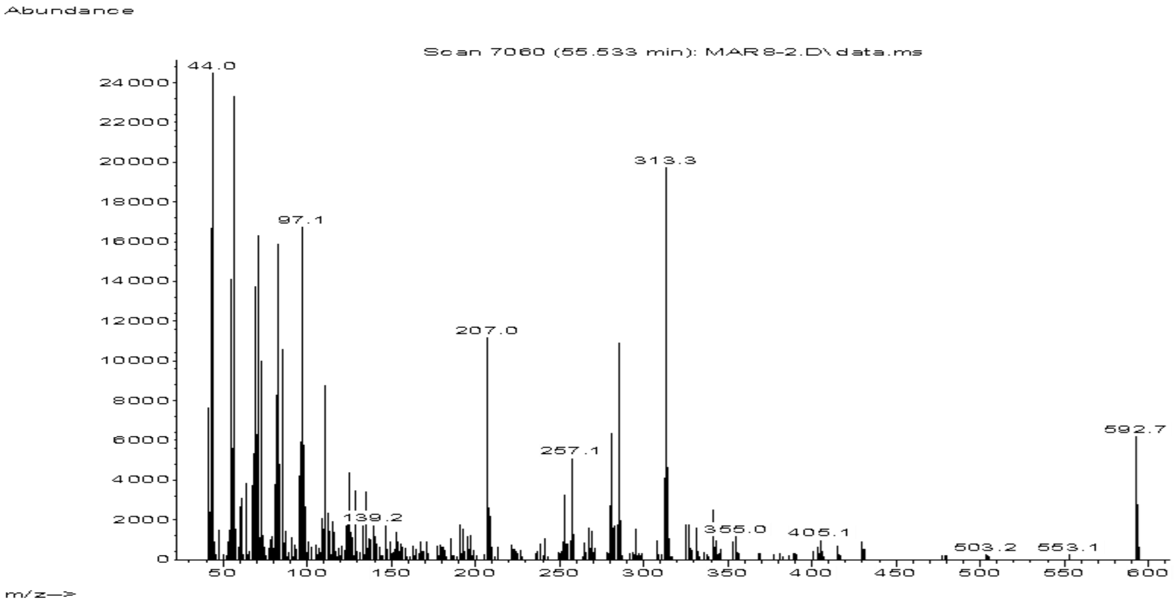

1

Fig. S2. Mass spectra of compounds **1-7.**

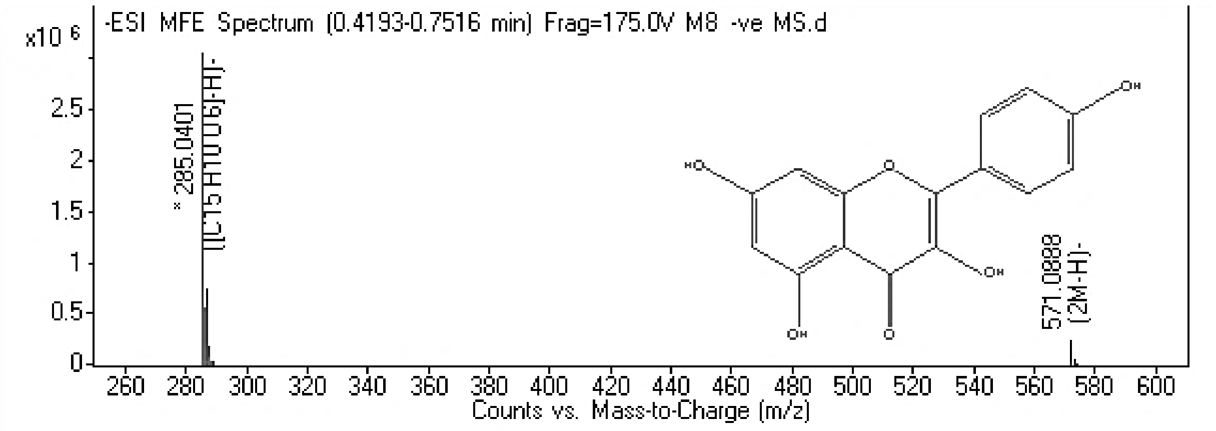

2

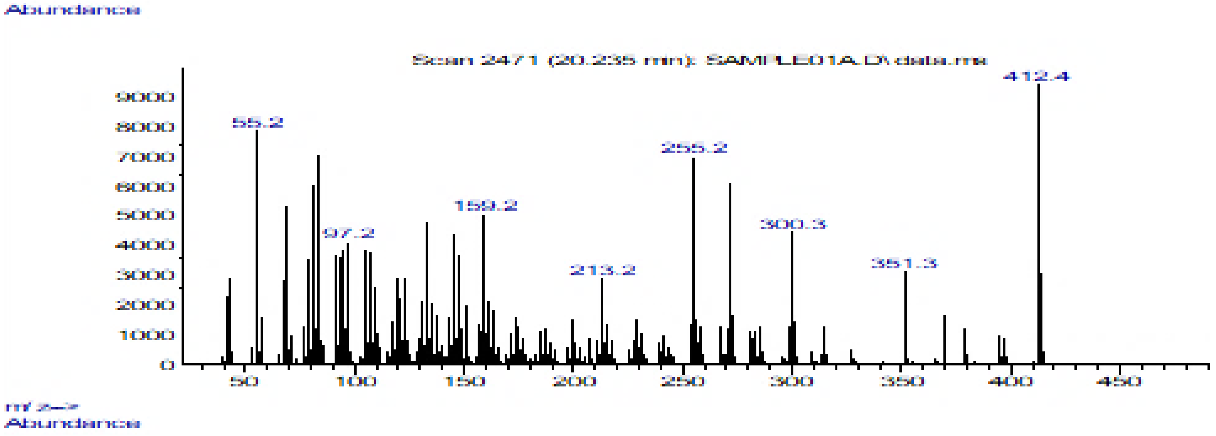

3

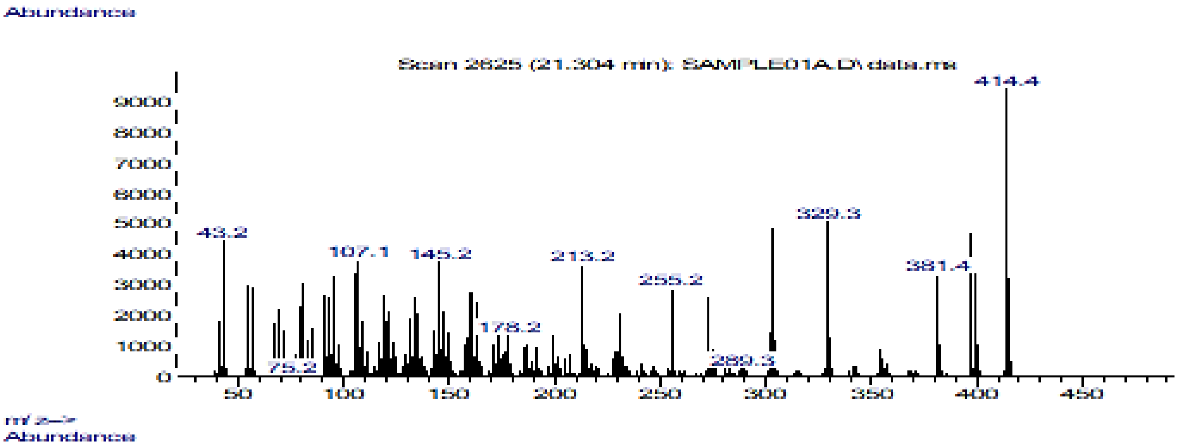

4

Cont. Fig. S2. Mass spectra of compounds **1-7.**

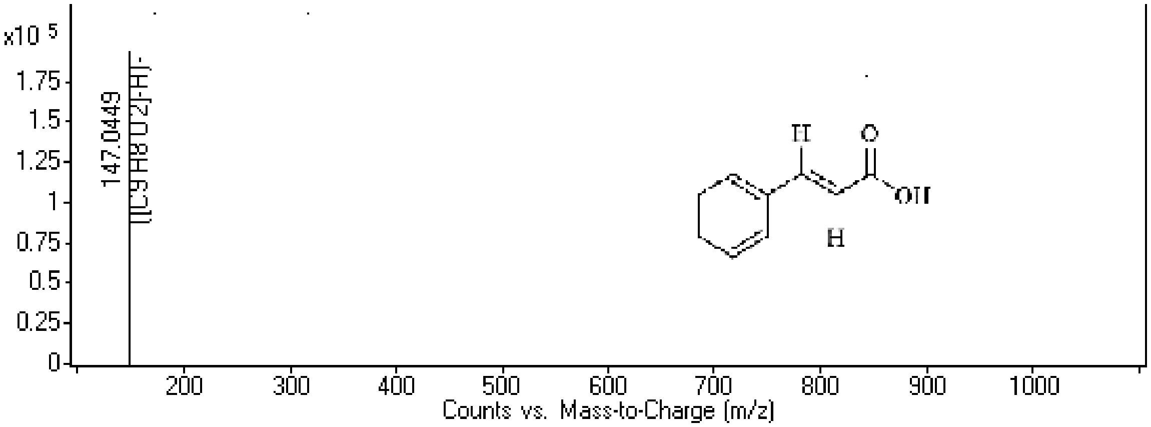

5

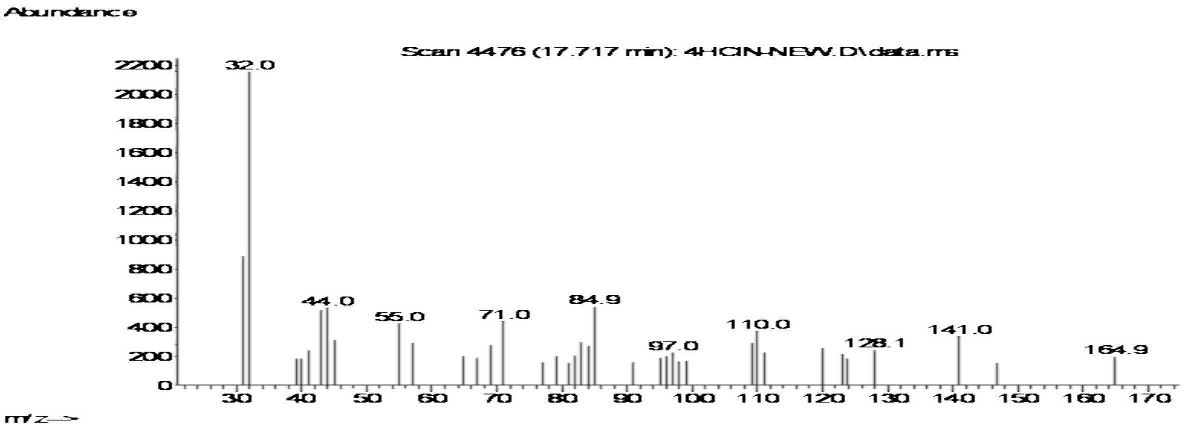

6

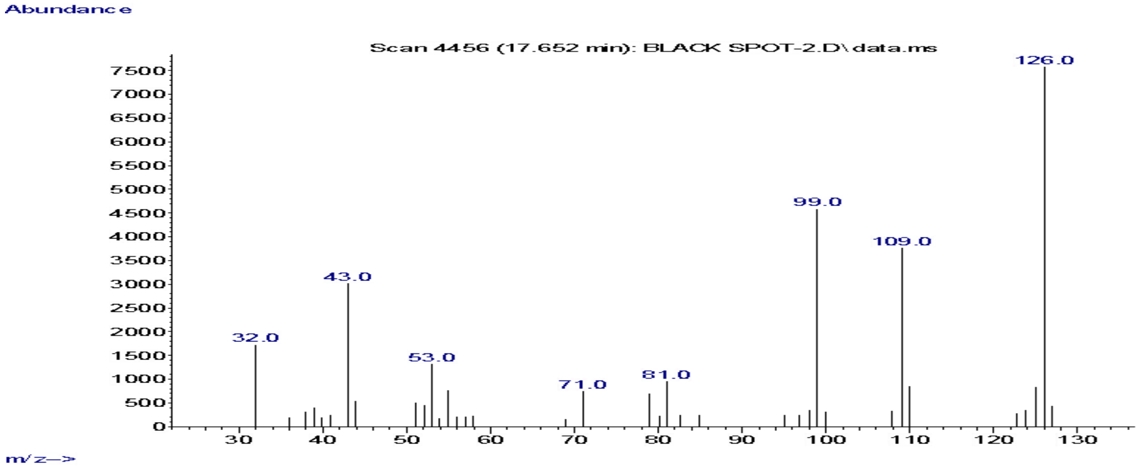

7

Cont. Fig. S2. Mass spectra of compounds **1-7.**

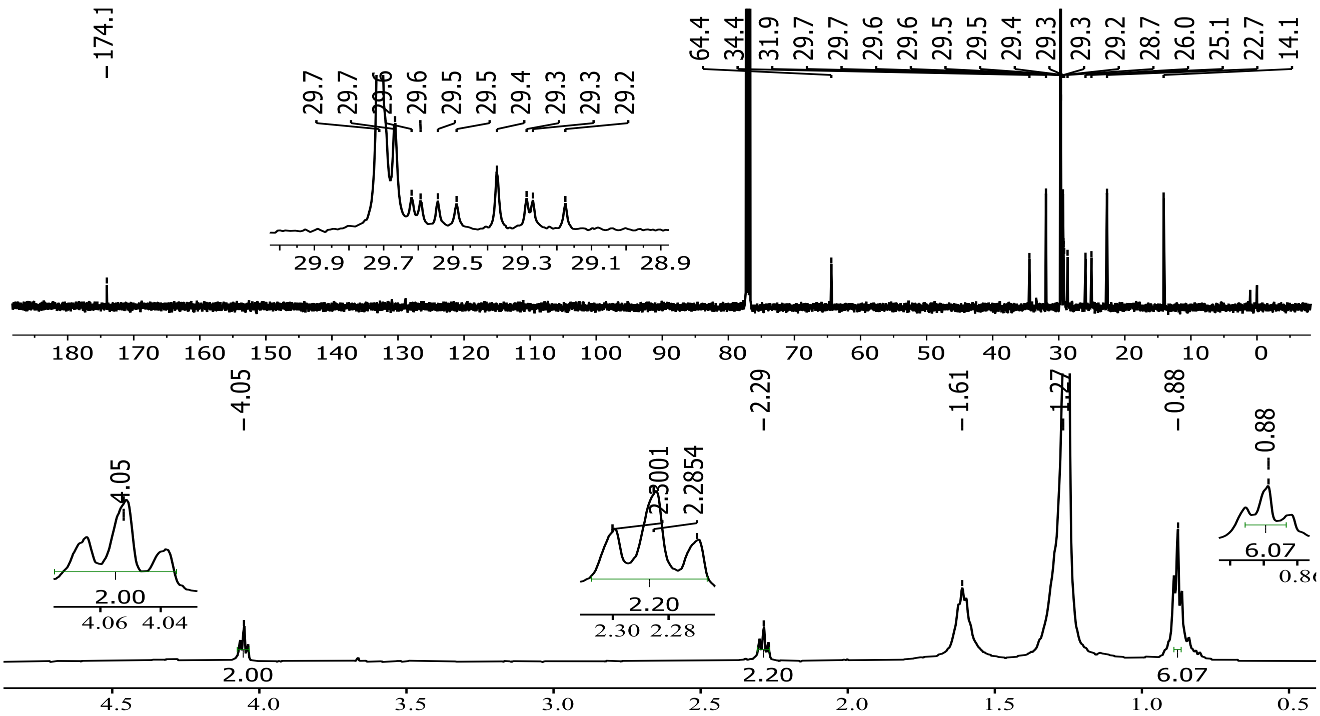

Figure S3. ^{1}H-NMR [$CDCl_3$, 500MHz] and ^{13}C-NMR of [$CDCl_3$, 125 MHz] of compound **1**.

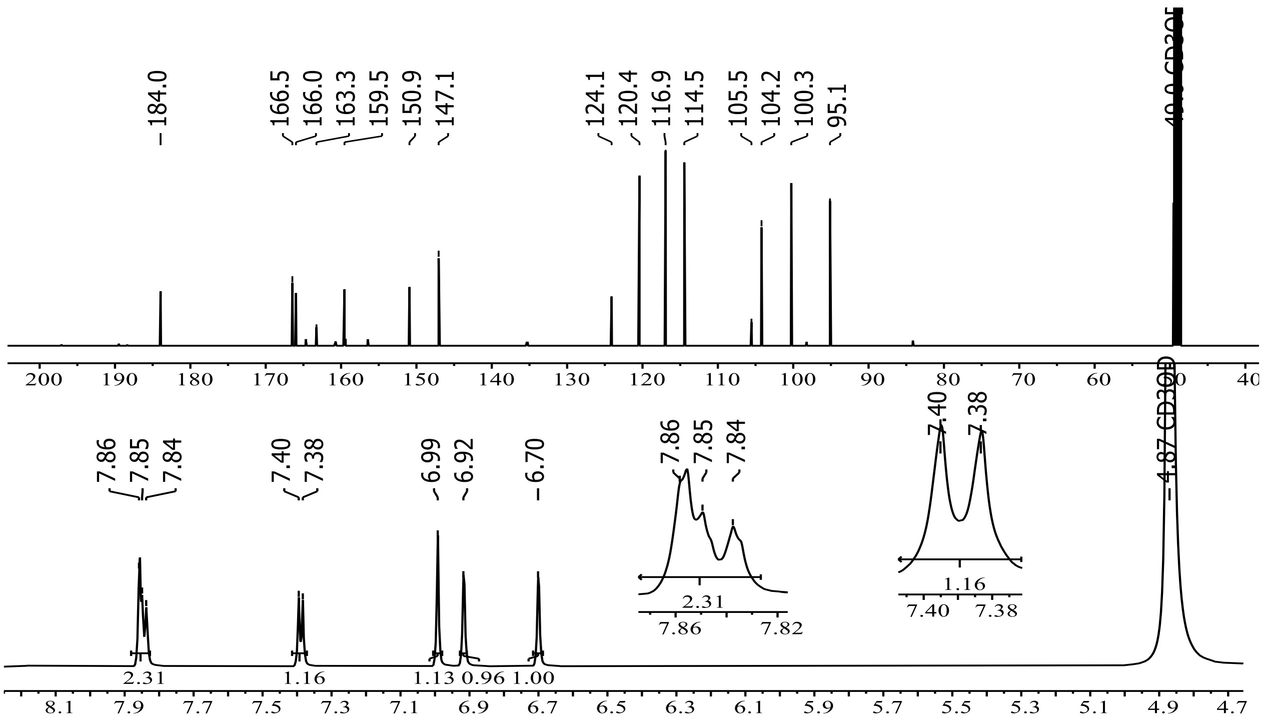

Figure S4. ^{1}H-NMR [CDCl3, 700MHz] and ^{13}C-NMR of [CDCl3, 175 MHz] of **2**.

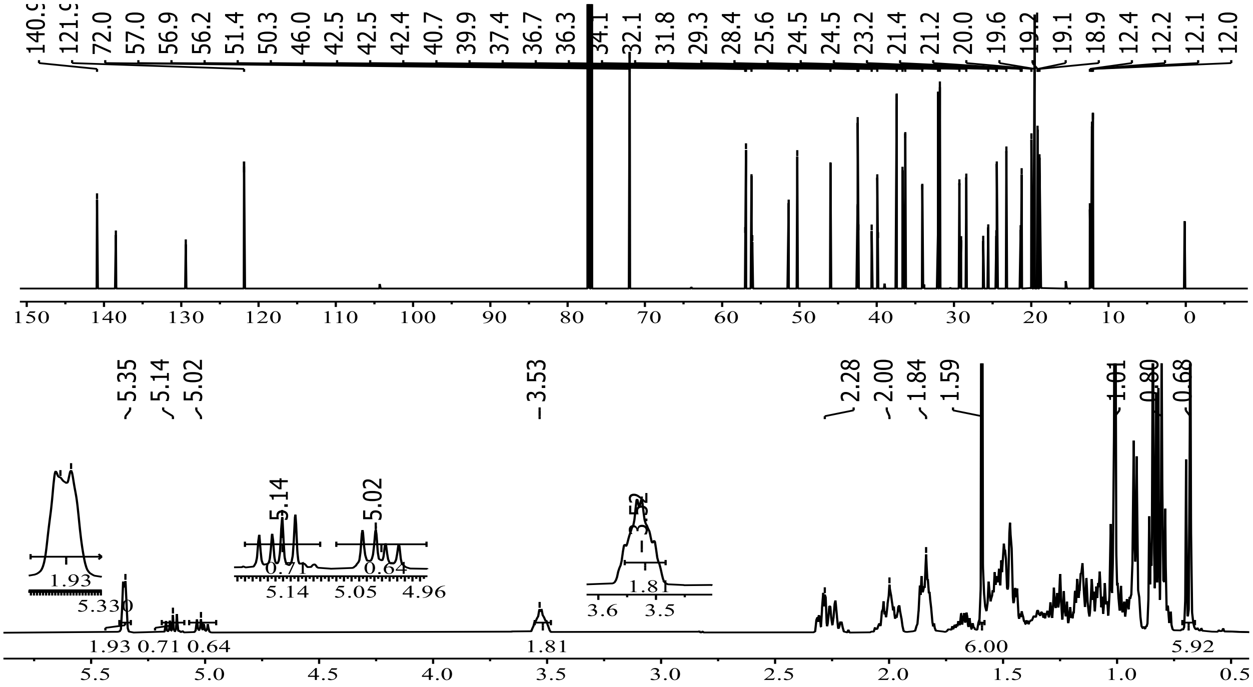

Figure S5. ^{1}H-NMR [$CDCl_3$, 500MHz] and ^{13}C-NMR of [$CDCl_3$, 125 MHz] of mixture of **3** and **4**.

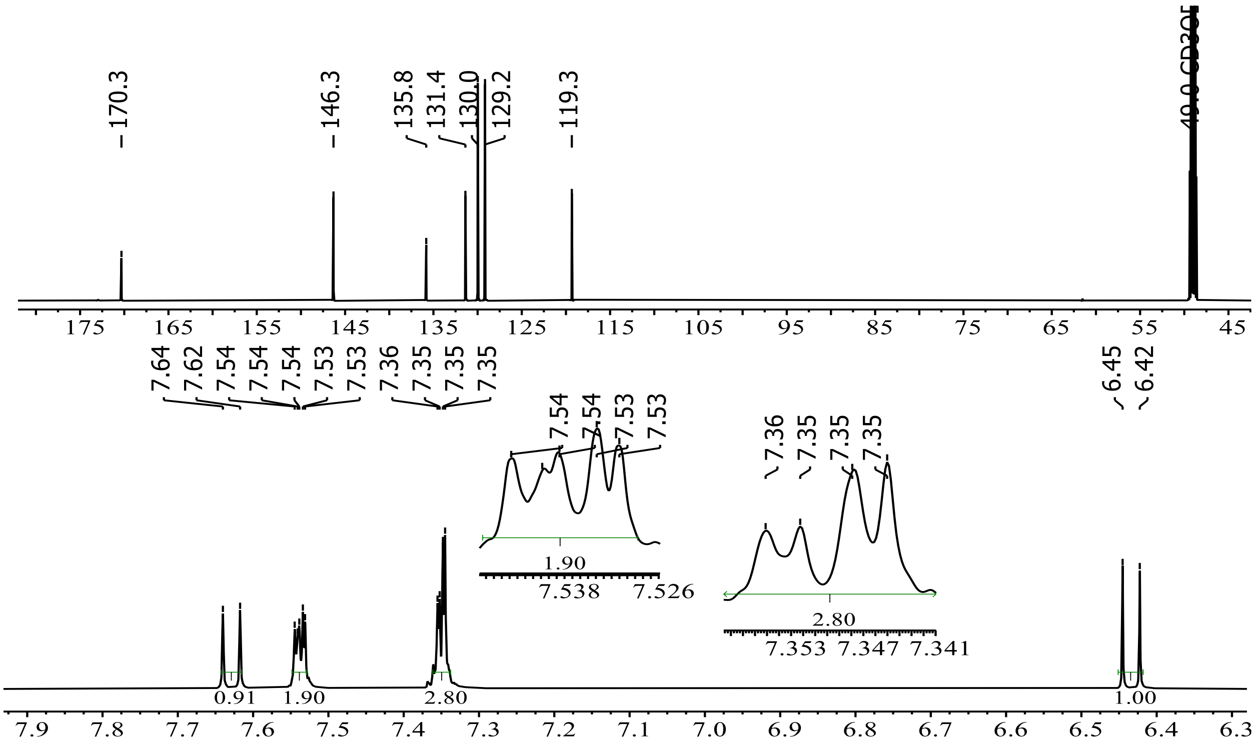

Figure S6. ^{1}H-NMR [CDCl3, 700MHz] and ^{13}C-NMR of [CDCl3, 175 MHz] of **5.**

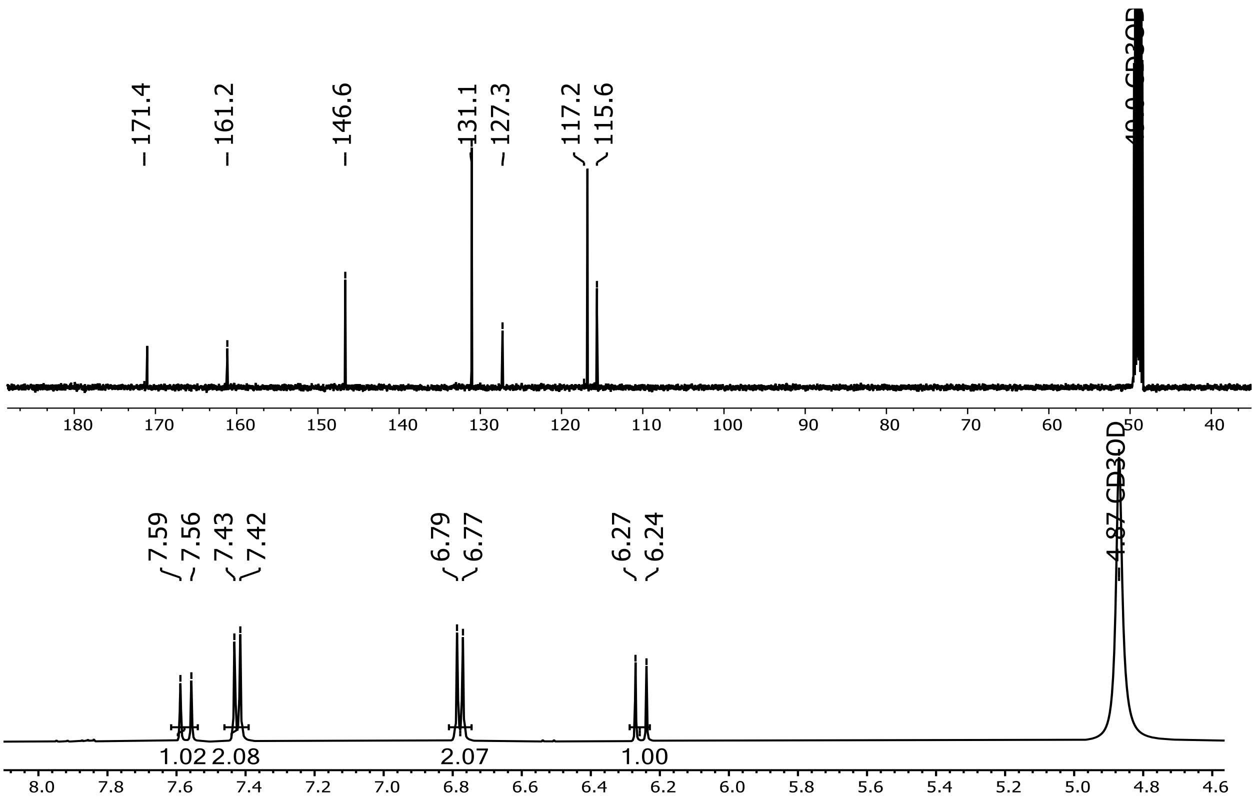

Figure S7. ^{1}H-NMR [CD_3OD, 700MHz] and ^{13}C-NMR of [CD_3OD, 175 MHz] of **6**.

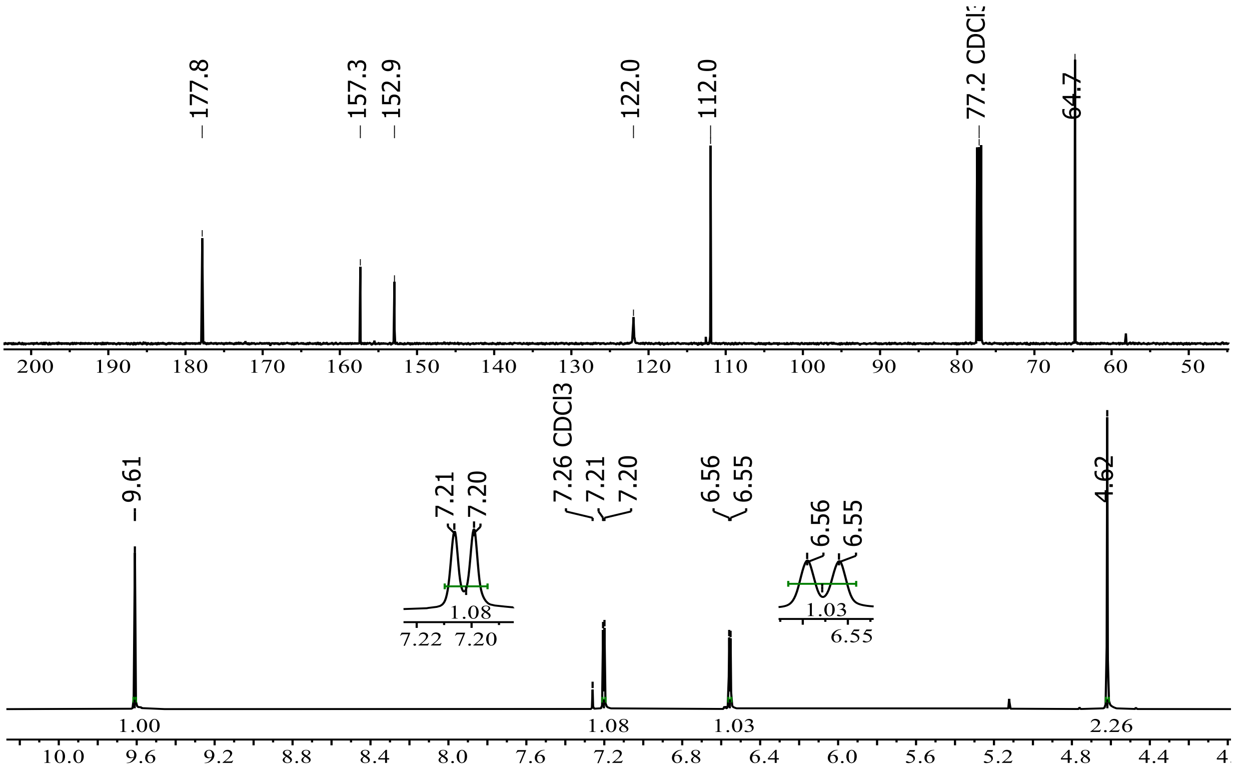

Figure S8. ^{1}H-NMR [CDCL3, 500MHz] and ^{13}C-NMR of [CDCL3, 125 MHz] of**7**.

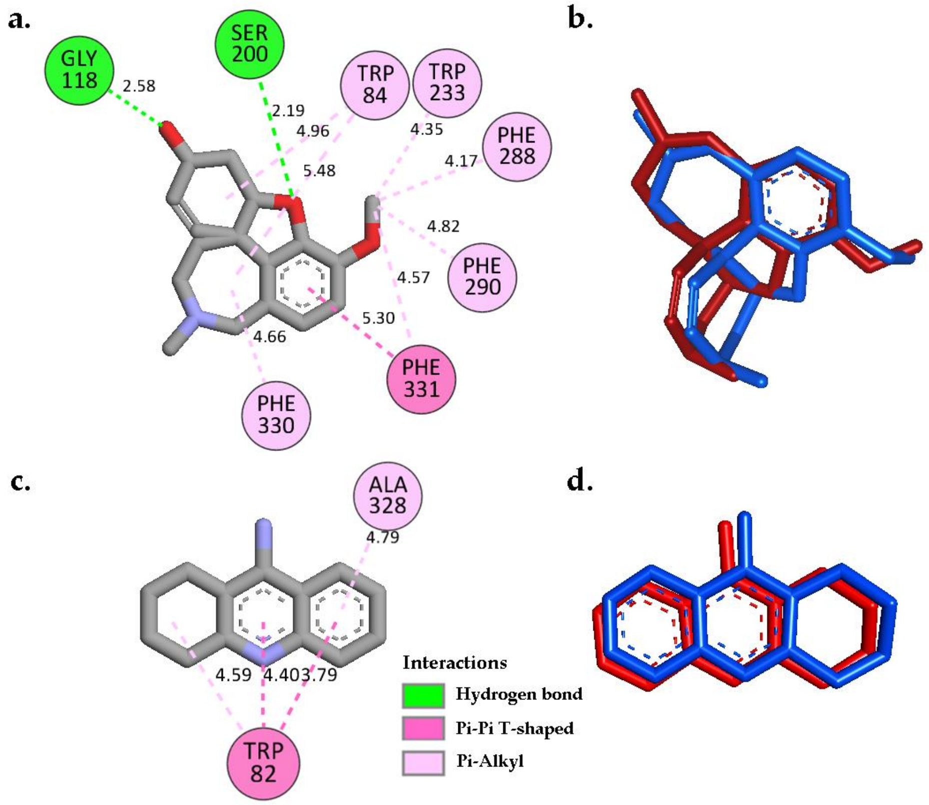

Figure S9. 2D-Molecular interaction binding models, as well the superimposed co-crystallized structural pose (blue) with the docked structure (red) of *Tc*AChE-galantamine derivative (a and b) with RMSD = 0.72 Å. And (c and d) for *Hs*BChE-tacrine (RMSD = 1.32 Å).

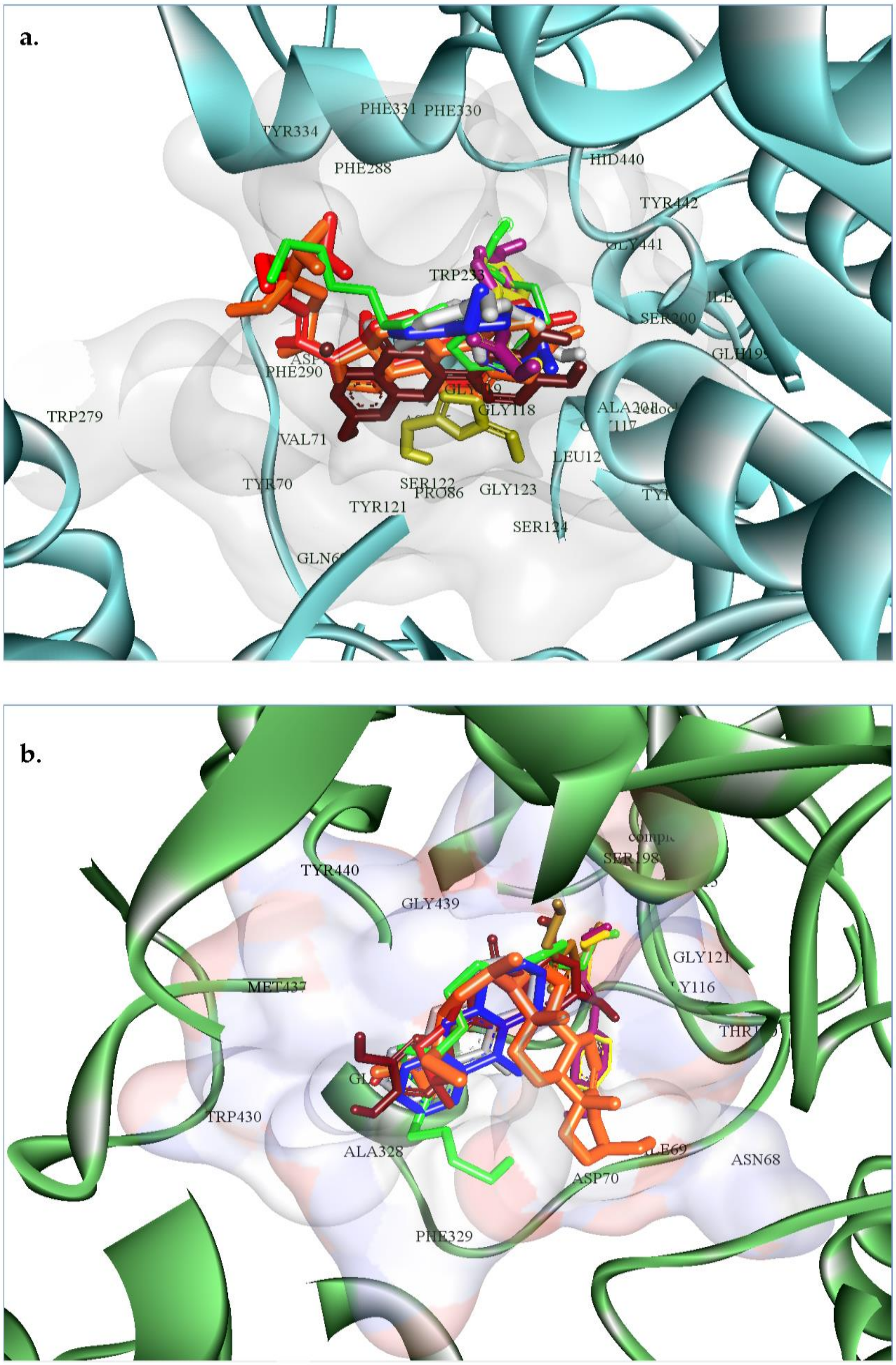

Figure S10. All docked compounds **1** (green), **2** (brown), **3** (orange), **4** (red), **5** (yellow), **6** (purple), **7** (gold), and the *in vitro* assay control (galantamine (grey)) were superimposed into the binding sites of *Tc*AChE (PDB ID: 1W6R) (a) and *Hs*BChE (PDB ID: 4BDS) (b). The co-crystalized ligands for *Tc*AChE (galantamine derivative) and

*Hs*BChE (tacrine) are in blue colour. Parts of the ribbon structure were removed to improve visualization.

Table S1. Interaction analysis for galantamine and chosen compounds **2**- **6** after docking into *Tc*AChE and *Hs*BChE binding sites.

**Ligand**	Enzyme	Binding site	Residue	Interaction	Distance (Å)	Ligand interacting moiety
**Galantamine**	*Tc*AChE	Catalytic tried	SER200	H-bond	2.15	Tetrahydrofuran ring
				H-bond	2.40	Methoxy
			HIS440	H-bond	2.70	Methoxy
				H-bond	2.73	Tetrahydrofuran ring
			TRP84	Hydrophobic	6.42	Cyclohexene ring
		Anionic site	PHE330	Hydrophobic	5.10	Cycloheptane ring
			PHE331	Hydrophobic	5.00	Aromatic ring
			GLY118	Hydrophobic	4.03	Aromatic ring
		Oxyanion hole	PHE288	Hydrophobic	4.66	C17
			PHE290	Hydrophobic	4.60	C17
		Acyl pocket	GLU199	H-bond	1.72	Hydroxyl at C10
			TRP233	Hydrophobic	4.47	C17
		Another region				
	HsBChE	Catalytic tried	SER198	H-bond	2.01	Tetrahydrofuran ring
			HIS438	H-bond	2.18	Tetrahydrofuran ring
			TRP82	Hydrophobic	3.88	Cycloheptane ring
		Anionic site		Hydrophobic	4.70	Cyclohexene ring
				Hydrophobic	5.30	C16
			TYR128	H-bond	1.98	Hydroxyl at C10
			PHE329	Hydrophobic	4.60	C17
			PHE398	Hydrophobic	4.66	C17
		Another region				
**2**	*TcAChE*	Anionic site	TRP84	Hydrophobic	4.47	Cyclohexadiene ring
		PAS	TYR121	H-bond	2.05	Hydroxyl at C2
				Hydrophobic	5.40	Aromatic ring
		Another region	ASN85	H-bond	1.73	Hydroxyl at C7
			GLY117	H-bond	2.89	Hydroxyl at C13
			GLU199	H-bond	1.85	Hydroxyl at C13
				H-bond	1.85	Hydroxyl at C14
	HsBChE	Catalytic tried	HIS438	H-bond	2.05	Hydroxyl at C14
			TRP82	Hydrophobic	4.20	Aromatic ring
		Anionic site		Hydrophobic	4.37	Cyclohexadiene ring
			GLY115	H-bond	2.74	Hydroxyl at C5
			GLU197	H-bond	2.19	Hydroxyl at C5

		Another region				
**3**	*TcAChE*	Anionic site	TRP84	Hydrophobic	3.82	C18
				Hydrophobic	4.30	Cyclohexene ring
				Hydrophobic	6.24	Cyclohexane ring
			PHE330	Hydrophobic	5.46	Cyclohexane ring
			PHE331	Hydrophobic	5.33	C24
		PAS	TYR70	Hydrophobic	4.52	C29
			TYR121	Hydrophobic	5.15	Cyclopentane ring
				Hydrophobic	5.15	C27
				Hydrophobic	5.18	Cyclohexane ring
			TRP279	Hydrophobic	4.44	C27
			TYR334	Hydrophobic	5.13	C24
		Another region	GLU199	H-bond	2.35	Hydroxyl at C2
	HsBChE	Anionic site	TRP82	Hydrophobic	4.16	Cycloheptane ring
				Hydrophobic	4.91	C24
		Another region	ASN68	H-bond	2.31	Hydroxyl at C2
			ALA328	Hydrophobic	3.49	C27
			TYR440	Hydrophobic	5.41	C24
**4**	*TcAChE*	Anionic site	TRP84	Hydrophobic	3.81	Cyclohexene ring
				Hydrophobic	3.93	C18
				Hydrophobic	6.06	Cyclohexane ring
			PHE330	Hydrophobic	4.58	C18
				Hydrophobic	4.75	C24
			PHE331	Hydrophobic	4.96	C24
		PAS	TYR70	Hydrophobic	4.73	C29
			TYR121	Hydrophobic	5.18	Cyclopentane ring
			TYR334	Hydrophobic	4.50	C24
				Hydrophobic	5.44	C29
		Another region	GLU199	H-bond	3.06	Hydroxyl at C2
	HsBChE	Anionic site	TRP82	Hydrophobic	4.15	Cyclopentane ring
				Hydrophobic	5.11	C27
		Another region	ASN68	H-bond	2.55	Hydroxyl at C2
			ALA328	Hydrophobic	3.40	C24
			TRP430	Hydrophobic	5.14	C27
			MET	Hydrophobic	5.29	C27
			TYR440	Hydrophobic	4.59	C27

**5**	*TcAChE*	Catalytic tried	HIS440	Hydrophobic	4.88	Aromatic ring
			TRP84	Hydrophobic	4.98	Aromatic ring
		Anionic site	PHE330	Hydrophobic	4.66	Aromatic ring
		Oxyanion hole	GLY118	H-bond	1.77	Hydroxy at carboxylate
	HsBChE	Anionic site	TYR128	H-bond	2.00	Carboxylate
		Another region	GLY115	H-bond	1.77	Carboxylate
			THR122	H-bond	2.18	Carboxylate
**6**	*TcAChE*	Catalytic tried	HIS440	H-bond	2.09	Hydroxyl at C1
				Hydrophobic	4.85	Cyclohexadiene ring
			TRP84	H-bond	3.04	Hydroxyl at C1
		Anionic site		Hydrophobic	5.20	Cyclohexadiene ring
			PHE330	Hydrophobic	4.46	Cyclohexadiene ring
		Oxyanion hole	GLY118	H-bond	1.79	Carboxylic acid
	HsBChE	Another region	ASN83	H-bond	1.96	Hydroxyl at C1
			GLY115	H-bond	1.84	Carboxylic acid
			THR122	H-bond	2.19	Carboxylic acid
